# Unveiling a Drift Resistant Cryptotope within *Marburgvirus* Nucleoprotein Recognized by Llama Single-Domain Antibodies

**DOI:** 10.3389/fimmu.2017.01234

**Published:** 2017-10-02

**Authors:** John Anthony Garza, Alexander Bryan Taylor, Laura Jo Sherwood, Peter John Hart, Andrew Hayhurst

**Affiliations:** ^1^Department of Virology and Immunology, Texas Biomedical Research Institute, San Antonio, TX, United States; ^2^X-Ray Crystallography Core Laboratory, Department of Biochemistry and Structural Biology, Institutional Research Cores, University of Texas Health Science Center at San Antonio, San Antonio, TX, United States; ^3^Department of Veterans Affairs, South Texas Veterans Health Care System, San Antonio, TX, United States

**Keywords:** filovirus, sdAb, VHH, nucleoprotein, crystallization chaperone, luciferase, Marburg, Ebola

## Abstract

Marburg virus (MARV) is a highly lethal hemorrhagic fever virus that is increasingly re-emerging in Africa, has been imported to both Europe and the US, and is also a Tier 1 bioterror threat. As a negative sense RNA virus, MARV has error prone replication which can yield progeny capable of evading countermeasures. To evaluate this vulnerability, we sought to determine the epitopes of 4 llama single-domain antibodies (sdAbs or VHH) specific for nucleoprotein (NP), each capable of forming MARV monoclonal affinity reagent sandwich assays. Here, we show that all sdAb bound the C-terminal region of NP, which was produced recombinantly to derive X-ray crystal structures of the three best performing antibody-antigen complexes. The common epitope is a trio of alpha helices that form a novel asymmetric basin-like depression that accommodates each sdAb paratope *via* substantial complementarity-determining region (CDR) restructuring. Shared core contacts were complemented by unique accessory contacts on the sides and overlooks of the basin yielding very different approach routes for each sdAb to bind the antigen. The C-terminal region of MARV NP was unable to be crystallized alone and required engagement with sdAb to form crystals suggesting the antibodies acted as crystallization chaperones. While gross structural homology is apparent between the two most conserved helices of MARV and *Ebolavirus*, the positions and morphologies of the resulting basins were markedly different. Naturally occurring amino acid variations occurring in bat and human *Marburgvirus* strains all mapped to surfaces distant from the predicted sdAb contacts suggesting a vital role for the NP interface in virus replication. As an essential internal structural component potentially interfacing with a partner protein it is likely the C-terminal epitope remains hidden or “cryptic” until virion disruption occurs. Conservation of this epitope over 50 years of *Marburgvirus* evolution should make these sdAb useful foundations for diagnostics and therapeutics resistant to drift.

## Introduction

Marburg virus (MARV) is a single-stranded, negative-sense RNA virus, which first emerged almost half a century ago in Europe to cause transmissible hemorrhagic fever in vaccine production staff handling African green monkey tissues imported from Uganda (1). Reservoiring in Egyptian fruit bats (*Rousettus aegyptiacus*), which are native to large regions of Africa (2), MARV has re-emerged to spill over into human populations sporadically with increasing severity (3–5). With no approved vaccines or therapeutics available, though several in development (6), diagnosis, quarantine, and contact-tracing have been effective at containing outbreaks so far (5, 7). However, as seen recently in West Africa with the related filovirus *Ebolavirus* (EBOV), outbreaks in highly mobile and populated areas can be difficult to extinguish, especially when combined with limited healthcare infrastructures (8).

Compared to other negative-strand RNA viruses such as influenza A, filoviruses appear relatively stable between different years and geographies, suggesting a high degree of adaptation to the reservoir host(s). However, where extensive human to human transmission has occurred across West Africa, mild viral evolution is apparent for EBOV with mutations improving viral fitness being recently defined (9). Though MARV outbreaks have so far been much smaller, with less extensive human to human transmission sometimes involving multiple separate spillovers, genomic variation has been observed in the largest outbreaks that occurred in Angola and Democratic Republic of Congo (DRC) (4). Nucleotide changes can impact the performance of sequence-based therapeutics (10) and diagnostics assays (11), making it imperative to keep such countermeasures up to date with currently circulating strains (12). Non-synonymous nucleotide changes can also alter the performance of virus protein-based therapeutics (10), especially those targeting the glycoprotein (GP), since it is the target for neutralizing antibodies generated by the host humoral response. Antibodies cloned from human survivors (13) and murine hybridomas (14) can all select escape mutants *in vitro* for MARV GP, which parallels the situation for EBOV as shown *in vitro* (15, 16) and *in vivo* (17), indicating a high degree of epitope plasticity for GP. Though internal viral antigens are not known to be overtly subject to antibody based immune surveillance, they are subject to T-cell surveillance which can cause selection of T-cell epitope variants. Such variations along with enabling compensatory, stabilizing (18), and random mutations can impact sequence (19) and protein-targeted countermeasures.

With these factors in mind, a single monoclonal affinity reagent may at first appear risky as the foundation for long-term viral recognition. However, we postulate that carefully selected non-neutralizing binders to highly conserved motifs of internal antigens should virtually eliminate the chances of antibody reactivity being diminished by natural viral evolution. Previously, we had selected four unique sdAb specific to MARV by panning our semi-synthetic phage display library (20) on virus preparations at BSL-4 (21). Each sdAb recognized nucleoprotein (NP), a critical viral structural protein enveloping the RNA genome as part of the viral ribonucleocapsid (22) and also a vital component of the viral assembly (23) and replication machineries in concert with VP35, polymerase L (24), and VP30 (25). The sdAbs were capable of sensitive and specific recognition of MARV Musoke and Angola strains, plus the closely related Ravn virus (RAVV) in a monoclonal affinity reagent sandwich assay, where a single antibody acts as both captor and tracer against polyvalent antigen (26, 27). Wishing to advance these sdAb further as diagnostic and transbody-based countermeasures, it was imperative that we find out precisely where and how they bind NP, to gauge the likely impact of MARV and RAVV evolution on the sdAb–NP recognition process. To pursue antibodies that are vulnerable to epitope erosion would be foolhardy in the long term, yet to identify antibodies that target completely conserved epitopes would be advantageous.

Here, using mutagenesis and X-ray crystallography, we determine the region of NP recognized by the sdAb and, in so doing, discover a novel tertiary structure of MARV NP. Elucidating this cryptic epitope or “cryptotope” allowed us to predict the likely impact bat and human MARV variation might have upon sdAb interactions, allowed us to compare and contrast local MARV and EBOV NP structures, and speculate on its natural role in viral replication.

## Results

### Anti-MARV sdAb Bind the NP C-Terminus with nM EC_50_ and Differing Conformational Sensitivities

**Figure 1:**
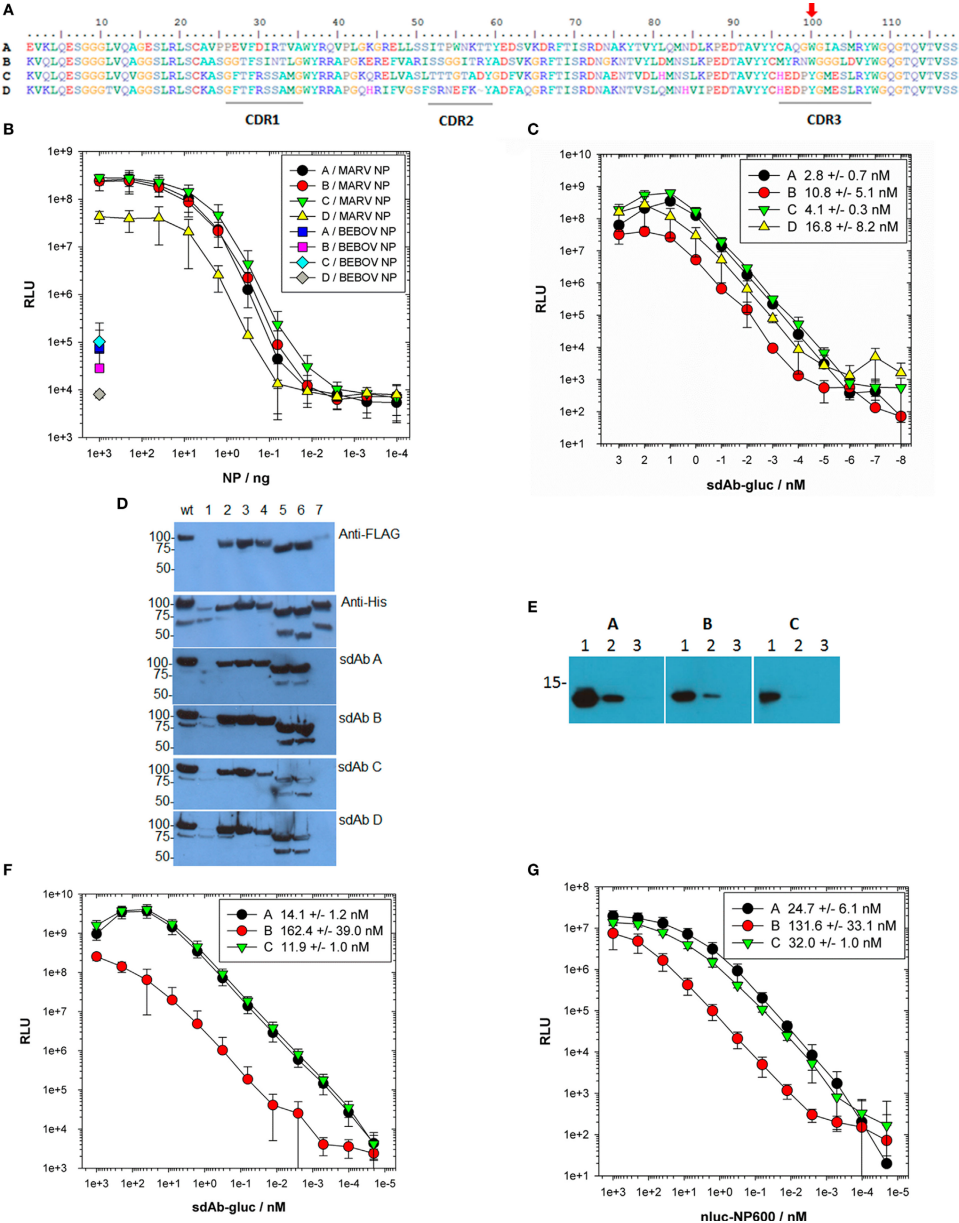
Locating the region of nucleoprotein (NP) bound by sdAb and establishing EC_50_ values. **(A)** Predicted amino acid sequences of the 4 anti-Marburg virus (MARV) NP–sdAb made using BioEdit (29) with complementarity-determining regions (CDRs) underlined and the aromatic residue in CDR3 highlighted with a red arrow. **(B)** Purified recombinant MARV or Bundibugyo (BEBOV) NP polymers were titrated over passively immobilized anti-MARV NP–sdAb, and captured NP subsequently detected with a constant amount of each phage displayed sdAb followed by anti-M13–horseradish peroxidase (HRP) conjugate. The experiment was performed three times in duplicate wells and the plots represent the mean values with error bars representing ± SD. **(C)** Fusions of sdAb–gluc were titrated over passively immobilized MARV NP polymer to determine EC_50_ values. Each titration was performed in duplicate wells with a negative control binding curve on BEBOV NP subtracted from each MARV curve. Each experiment was repeated three times and the plots represent the mean values with error bars representing ± SD. The EC_50_ values were determined for each curve and are shown in the legend ± SD. **(D)** Lysates of *E. coli* expressing MARV NP tagged with FLAG at the N-terminus and His_6_ at the C-terminus were probed with either anti-FLAG-HRP, anti-His_6_-HRP or sdAb–alkaline phosphatase (AP) fusion proteins. Wt represents full-length NP while numbers refer to the deletions of amino acids; 1–100 (1), 101–200 (2), 201–300 (3), 301–400 (4), 401–500 (5), 501–600 (6), and 601–695 (7). **(E)** 1,000 ng (1), 100 ng (2), or 10 ng (3) of purified MARV NP C-terminus was probed with 100 nM of each sdAb–AP fusion protein for equivalent times and developed side-by-side. **(F)** The sdAb–gluc fusions were titrated over passively immobilized mbp-NP600 fusion protein to determine the monovalent EC_50_ values. Each titration was performed in duplicate wells with a negative control mbp only binding curve subtracted from each mbp-NP600 curve. The experiment was repeated three times and the plots represent the mean values with error bars ± SD. The EC_50_ values were determined for each curve and are shown in the legend for each sdAb–gluc fusion protein ± SD. **(G)** The nluc-NP600 fusions were titrated over oriented immobilized sdAb to determine the EC_50_ values in a reversed orientation. Each titration was performed in duplicate wells with a negative control nluc only binding curve subtracted from each nluc-NP600 curve. The experiment was repeated three times and the plots represent the mean values with error bars ± SD. The EC_50_ values were determined for each curve and are shown in the legend for each sdAb ± SD.

Predicted amino acid sequences of the four anti-MARV sdAbs (Figure 1A) reveal three unique families with sdAb C and D sharing complementarity-determining regions (CDRs) 1 and 3 and all four sdAbs sharing an aromatic residue in the middle of CDR3. Sandwich-based detection of Triton lysed virus employing each purified sdAb as captor and phage displayed sdAb tracer (21) was recapitulated on purified NP (Figures S1A,B in Supplementary Material). The trend shown in Figure 1B suggested that other MARV ribonucleoprotein components were unlikely to be involved in sdAb binding in this semi-quantitative polyvalent antigen capture format. In this assay, sdAb D was re-confirmed as the poorest performing clone and was only sparingly studied further since it was also a relatively poor expresser. That sdAb D shares CDRs 1 and 3 with sdAb C yet appears to bind poorly suggest framework region (FR) residues or CDR2 composition might be non-optimal. Prior to any structural work, we mutated the single aromatic residue of CDR3 of sdAb A-C to alanine, and purified mutant proteins (Figure S2A in Supplementary Material) to explore the impact on binding NP since it is known that aromatic R-groups, especially in CDR3 perform critical antigen binding services (28). All three sdAb show diminished antigen binding when amino acid 100 was substituted for alanine (Figure S2B in Supplementary Material). Wild-type sdAb A and C are equivalent binders while sdAb B is a relatively poor performer in this format, where immobilized polyvalent NP captures monomeric sdAb which is then revealed with bivalent anti-His_6_ IgG horseradish peroxidase (HRP).

To begin more quantitatively ranking the sdAb, we engineered “glucibodies” which are fusions of sdAb to the N-terminus of *Gaussia* luciferase (gluc) (30). The gluc protein is a sensitive monomeric reporter enzyme and is efficiently secreted to the periplasmic compartment of *E. coli* when fused to other types of recombinant antibody fragments (31) offering a facile route to determine sdAb EC_50_ value for each sdAb. Titration of purified sdAb–gluc fusion proteins (Figures S3A,B in Supplementary Material) over immobilized polyvalent NP defined the EC_50_ values for each antibody in the low nanomolar range (Figure 1C) though with no statistical difference (*P*-value > 0.05).

Deletion mutagenesis of *E. coli* expressed NP followed by probing with bivalent sdAb–alkaline phosphatase (AP) fusion proteins to leverage avidity and precipitating chemiluminescent product, revealed loss of binding for all four sdAb when the last 95 amino acids were absent (Figure 1D). Nineteen anti-Ebola virus sdAb previously isolated from the same phage displayed sdAb library using similar selections on four species of EBOV also recognized the NP C-terminal regions and performed as both captor and tracer (27), indicating a particularly attractive epitope for sdAb appears to reside here. The C-termini of both MARV and EBOV NP are known to be repetitively displayed along the ribonucleocapsid (22, 32), explaining why our anti-MARV sdAb perform akin to our anti-Ebola sdAb in sandwich immunoassays, where polyvalent antigen enables one sdAb clone to serve as both captor and tracer.

When the last 95 amino acids of MARV Musoke NP were overexpressed and purified as an N-terminally His-tagged motif (termed NP600) (Figures S4A–C in Supplementary Material), the isolated antigen was able to be recognized by the three bivalent sdAb–AP tested by Western blot, though sdAb C was a relatively poor binder (Figure 1E with original blots shown in Figure S5 in Supplementary Material). Since passively immobilized NP600 was also a poor substrate for sdAb C glucibody (data not shown), we immobilized purified fusion proteins (Figures S6A,B in Supplementary Material) of maltose-binding protein (mbp) and NP600 to determine monovalent EC_50_ values for the sdAb as glucibodies (Figure 1F). Single-domain antibody B glucibody was significantly different from both sdAb A and C glucibodies (*P*–value <0.05). To reconfirm these findings using solution phase NP600 antigen, we reversed the assay orientation by immobilizing sdAb *via* a single biotin acceptor peptide (BAP) on a neutravidin coat. The sdAb were probed with purified fusion proteins of NP600 to the C-terminus of nluc (Figures S7A,B in Supplementary Material), another small monomeric luciferase heavily engineered for brightness (33). The nluc protein is highly soluble in the cytosol of *E. coli*, though in our hands is poorly secreted to the periplasm, making it an ideal fusion partner for NP600 which we were also unable to secrete efficiently. Titration of nluc-NP600 fusions over the oriented sdAb revealed EC_50_ values for each antibody on par as before (Figure 1G), with sdAb B significantly different from sdAb A but not to sdAb C (*P*–value <0.05). The monovalent EC_50_ values for the sdAb in both assay formats were higher than when using polyvalent NP antigen as expected, yet the ranking of sdAb A followed by sdAb C and then sdAb B tended to be preserved.

Since linear peptide arrays representing the length of NP600 were unable to identify any reactivity with sdAb C (data not shown) and sdAb C reacted poorly with NP600 on Western blots indicated dependence on a conformational epitope. Classifying epitopes as conformational or non-conformational solely based on Western blotting is ill-advised as immunoblotted antigens can retain sufficient structural information for at least some binding by the sdAb (34). To define the epitope(s) further we chose X-ray crystallography, since it would also yield the structure for the MARV NP C-terminus which has so far proven elusive to tertiary structural assignment (35).

### Difficulty Generating Crystals of sdAb–Antigen Complexes Mirrored Reliance on Conformation

Both sdAb A and sdAb B were straightforward to crystallize alone, and in complex with NP600 simply by equilibrating an approximate 1:1 M ratios of the purified sdAb and NP600 proteins overnight before dispensing into crystallization screening experiments. However, sdAb C was highly refractory to crystallization alone and yielded a single polycrystalline cluster from thousands of screening trials. While further attempts to improve this crystal form were unsuccessful, the structure was determined and revealed the C-terminal His_6_ tag provided fortuitous packing interactions. We were unable to co-crystallize sdAb C with NP600 by simple equilibration after mixing or following size exclusion chromatography (SEC) of the complex. We, therefore, used a bait prey strategy to allow facile production and purification of large amounts of pre-formed antibody-antigen complexes. We removed the C-terminal His_6_ tag from sdAb C (prey), isolating it from crude osmotic shocks using partially purified His_6_-tagged NP600 (bait), and then employed immobilized metal affinity chromatography (IMAC) followed by SEC to purify the complex which yielded occasional, poorly diffracting small crystals. We repeated the strategy using a trimmed version of NP600 that begins at Trp632 (termed NP632), to avoid potentially flexible regions not visible in the sdAb A or B complex structures, resulting in pure sdAb C/NP632 complex (Figure S8 in Supplementary Material). Within the first screen, two wells with small, irregularly shaped, poorly diffracting crystals were discovered that, upon further optimization, yielded crystals that diffracted satisfactorily.

The EC_50_ of sdAb A, B, and C glucibodies for mbp-NP632 were determined to be 15.4 ± 3.9; 189.1 ± 55; 22.3 ± 3.2 nM, respectively, while EC_50_ values of nluc-NP632 for sdAb were 12.8 ± 4.3; 28.5 ± 3.6; 26.6 ± 3.4 nM, respectively (Figures S9A,B in Supplementary Material). In both cases, the sdAb B EC_50_ value was significantly different from those of sdAb A and sdAb C (*P*-value <0.05). The overall similarity between EC_50_ values determined using NP600 or NP632 suggests the first 31 amino acids of NP600 that are absent in NP632 are not critical for sdAb binding, though sdAb B exhibits variation depending on the assay format. We were unable to generate suitable crystals of NP600 alone, and NP632 proved somewhat insoluble unless produced as a fusion protein. To date, we have also been unable to generate crystals of mbp-NP600 or mbp-NP632, suggesting that our semi-synthetic sdAb had a chaperone effect on the ability of the C-terminal domain to crystallize, as seen previously for a protein refractory to crystallization by itself (36). X-ray diffraction data collection and statistics for the bound and unbound crystal structures for sdAb A–C are shown in Table 1.

**Table 1:** Data collection and refinement statistics.

	sdAb A	sdAb A/NP600	sdAb B	sdAb B/NP600	sdAb C	sdAb C/NP632
PDB code	6APO	6APP	6APQ	4W2O	4W2P	4W2Q
**Data collection**						
X-ray source	Advanced Photon Source 24-ID-E	UTHSCSA X-ray Crystallography Core Laboratory	Advanced Photon Source 24-ID-E	Advanced Light Source 4.2.2	Advanced Photon Source 24-ID-C	UTHSCSA X-ray Crystallography Core Laboratory
Space group	*P*2_1_2_1_2_1_	*P*2_1_2_1_2_1_	*P*6_5_22	*P*2_1_2_1_2_1_	*P*1	*P*2_1_
Cell dimensions						
*a, b, c* (Å)	41.6, 49.4, 58.8	41.6, 46.2, 102.8	80.0, 80.0, 89.9	58.0, 108.7, 141.3	33.4, 49.5, 65.4	57.7. 98.5, 68.5
α, β, γ, (°)	90, 90, 90	90, 90, 90	90, 90, 120	90, 90, 90	87.7, 84.8, 79.4	90, 96.2, 90
Wavelength (Å)	0.97917	1.54178	0.97917	0.97626	0.97950	1.54178
Resolution (Å)	41.60–1.17 (1.23–1.17)^a^	46.15–1.75 (1.84–1.75)	69.27–1.90 (2.00–1.90)	58.00–3.20 (3.37–3.20)	48.65–1.77 (1.86–1.77)	46.42–2.70 (2.85–2.70)
*R*_sym_	0.067 (0.282)	0.089 (0.640)	0.084 (0.587)	0.189 (0.705)	0.063 (0.390)	0.154 (0.673)
*R*_pim_	0.028 (0.198)	0.039 (0.281)	0.043 (0.304)	0.081 (0.297)	0.058 (0.332)	0.092 (0.398)
Mean *I*/σ*I*	17.6 (3.4)	14.1 (2.6)	12.2 (2.5)	10.6 (3.0)	8.6 (2.3)	8.2 (1.9)
Completeness (%)	95.7 (71.3)	99.2 (98.1)	99.9 (100)	100 (100)	91.6 (88.5)	99.0 (98.3)
Redundancy	6.6 (3.2)	6.9 (6.9)	5.5 (5.7)	7.2 (7.4)	2.5 (2.4)	3.7 (3.8)
Wilson value (Å^2^)	9.3	19.0	26.9	55.1	16.3	35.8
**Refinement**						
Resolution (Å)	37.81–1.17	34.34–1.75	69.27–1.90	54.33–3.20	26.77–1.77	41.68–2.70
No. reflections	39,980	20,434	13,899	15,287	36,633	20,587
*R*_work/_*R*_free_	0.148/0.173	0.171/0.219	0.185/0.216	0.227/0.285	0.165/0.203	0.220/0.274
No. atoms						
Protein	952	1,450	901	5,716	3,745	5,720
Ion	–	–	4 (Na^+^, 3 Cl^−^)	40 (8 ${\text{SO}}_{4}^{2-}$)	9 (Na^+^, 2 CH_3_COO^−^)	–
Water	176	321	138	–	379	104
B-factors (Å^2^)						
Protein	12.2	19.0	28.3	51.2	19.3	38.9
Ion	–	–	31.2	69.7	17.8	–
Water	28.8	31.0	40.3	–	27.5	28.4
R.m.s deviations						
Bond lengths (Å)	0.007	0.007	0.007	0.003	0.010	0.005
Bond angles (°)	0.967	1.090	1.106	0.557	1.034	0.971
Ramachandran plot						
Favored (%)	98.3	99.4	99.1	94.5	98.7	98.9
Allowed (%)	1.7	0.6	0.9	4.6	1.3	1.1
Outliers (%)	0.0	0.0	0.0	0.8	0.0	0.0

*^a^Highest resolution shell is shown in parentheses*.

### sdAb Employ Common and Unique Approaches to Engage the MARV NP C-Terminus

**Figure 2:**
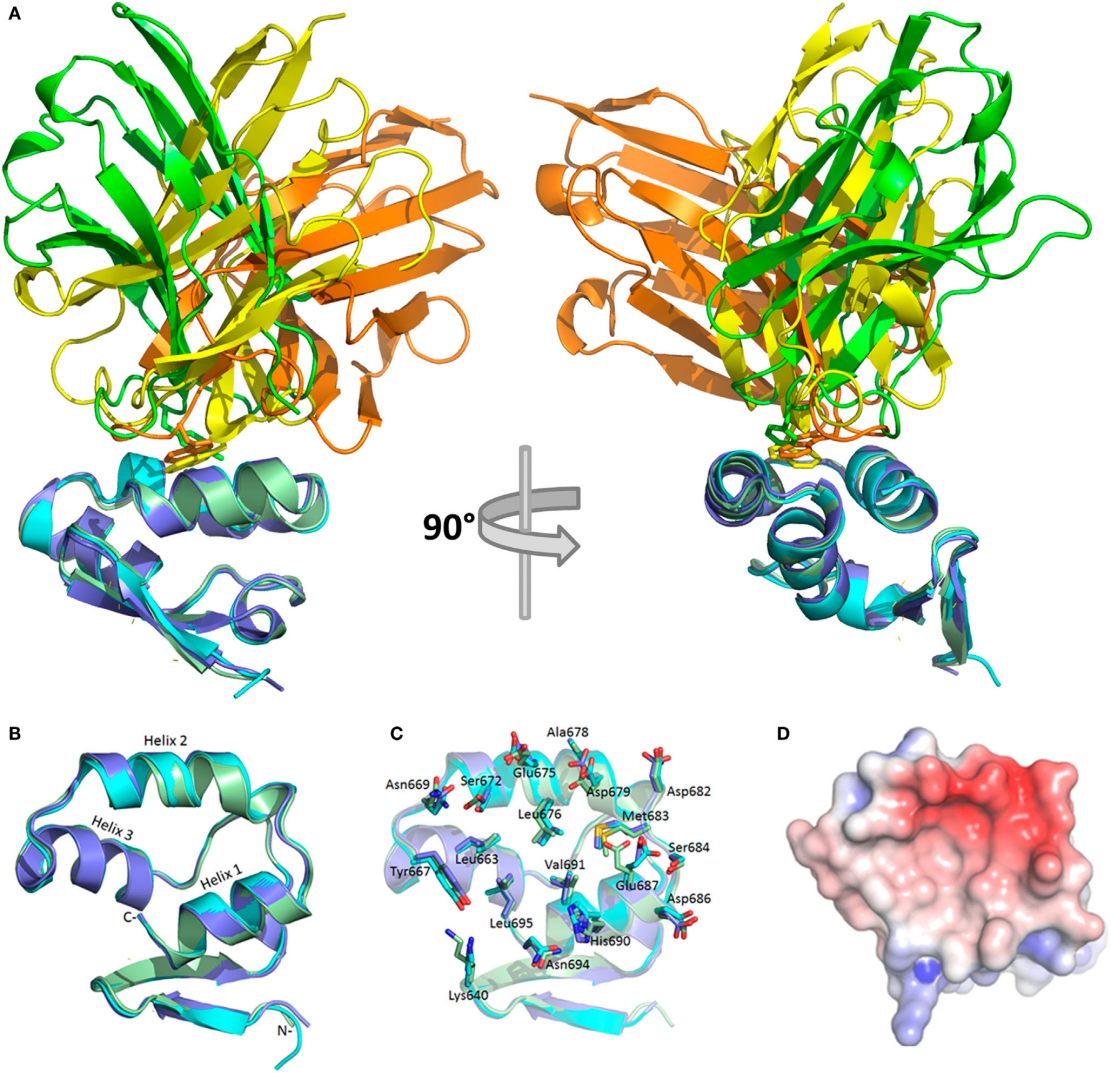
Crystal structures of bound sdAb A, B, and C and the cryptic Marburg virus (MARV) nucleoprotein (NP) C-terminus. **(A)** The three sdAb–NP complexes revealing the different approach angles for sdAb A (yellow), B (orange), and C (green). NP is colored for each sdAb thus; A (purple), B (pale green), C (cyan). NP is oriented to show helices 1 and 2 in the same plane while helix 3 descends at an approximate 45° angle left to right. Residues 600–631 of NP600 and the MetGlyHis_6_ tag are not visible in the crystal structures. Rotating the structures 90° reveals the end on view from the C-terminus of NP and the grouping of apical CDR3 aromatic residues shown as sticks between helices 1 and 2. **(B)** The sdAbs have been removed and the NP C-terminus rotated from its aspect in **(A)** to give a sdAb’s eye view of the epitope and the three major alpha helices with helix 1 (Leu695–Phe685) and helix 2 (Pro681–Pro670) forming a V-like shelf and basin sides, while helix 3 (Tyr667–Pro656) forms the basin floor. From the floor emerges anti-parallel beta-sheet that loops back around under helix 1 *via* Lys640 onward to Trp632. **(C)** NP residues implicated in engaging the sdAb have their side chains shown as sticks and are labeled. Almost all are shared by the three sdAb except Lys640 (not sdAb B), Val671 (not sdAb B or C), Val674 (not sdAb B or C), S684 (not sdAb A). **(D)** Electrostatic surface potential of the NP epitope with scale ranging from −5 (red) to +5 (blue) K_b_T/e_c_ reveals a broad asymmetric hydrophobic basin between helices 1 and 2 with an open aspect toward Tyr667 at the start of helix 3 and a closed aspect toward the descent with an overlook of negative charges in a crescent from Glu675 around to Glu687.

All three sdAb–NP complexes are shown in Figure 2A revealing the different approach angles used by the antibodies to interact with the MARV NP C-terminal domain with the pivotal CDR3 aromatic side chains shown in stick form. Unique VH and VL domains capable of binding the same epitope through overlapping but non-identical footprints resulting in different approach angles have been revealed to atomic resolution for broadly neutralizing IgG against viral envelope proteins of influenza A (37) and HIV-1 (38). Epitopes that can elicit a wide diversity of antibodies that are now able to be mined through various repertoire selections are dubbed supersites (39). A sdAb’s eye view of our more modest NP bijousite is shown in Figure 2B in cartoon form where the main chains of the three NP C-termini overlay with one another within 0.4–0.7 Å RMSD for all NP structures in the crystallographic asymmetric units. The last 64 residues of NP visible in the crystal structures primarily consist of three alpha helices associating to form an upper V-like shelf of the two C-terminal most helices (arbitrarily named 1 and 2 counting back from Leu695), with the third descending between them to re-appear after a turn as beta sheet positioned under the C-terminus. Contact mapping analysis using the Weizmann server running part of the SPACE suite (40) identified NP residues potentially involved in binding each sdAb with different combinations of CDRs engaging the three helices (Figure S10 in Supplementary Material). When side chains of all of the potential sdAb contacts are displayed as sticks on the epitope backbone (Figure 2C), minor differences are apparent in the disposition of R-groups (e.g., Asn694 and Glu687), though the epitope appears fairly constrained. Electrostatic surface rendering (Figure 2D) reveals an asymmetric basin-like depression between helices 1 and 2 with helix 3 forming the basin floor with a hydrophobic core of Leu676, Val691, and Met683 at the closed end, while Leu663, Leu695, and Tyr667 reside at the upper more open end. Single-domain antibodies are well known to target concave active sites of enzymes (41), recessed epitopes of parasite variant surface GPs (42), and canyons of virus particles (43), and it appears that the MARV NP C-terminal basin also constitutes such an attractive cryptotope. The basin overlook also offers potential for alternative modes of interaction with a crescent of negative charges (Glu675, Asp679, Asp682, Glu687, and Asp686) toward the closed end being noteworthy for salt bridge potential.

**Figure 3:**
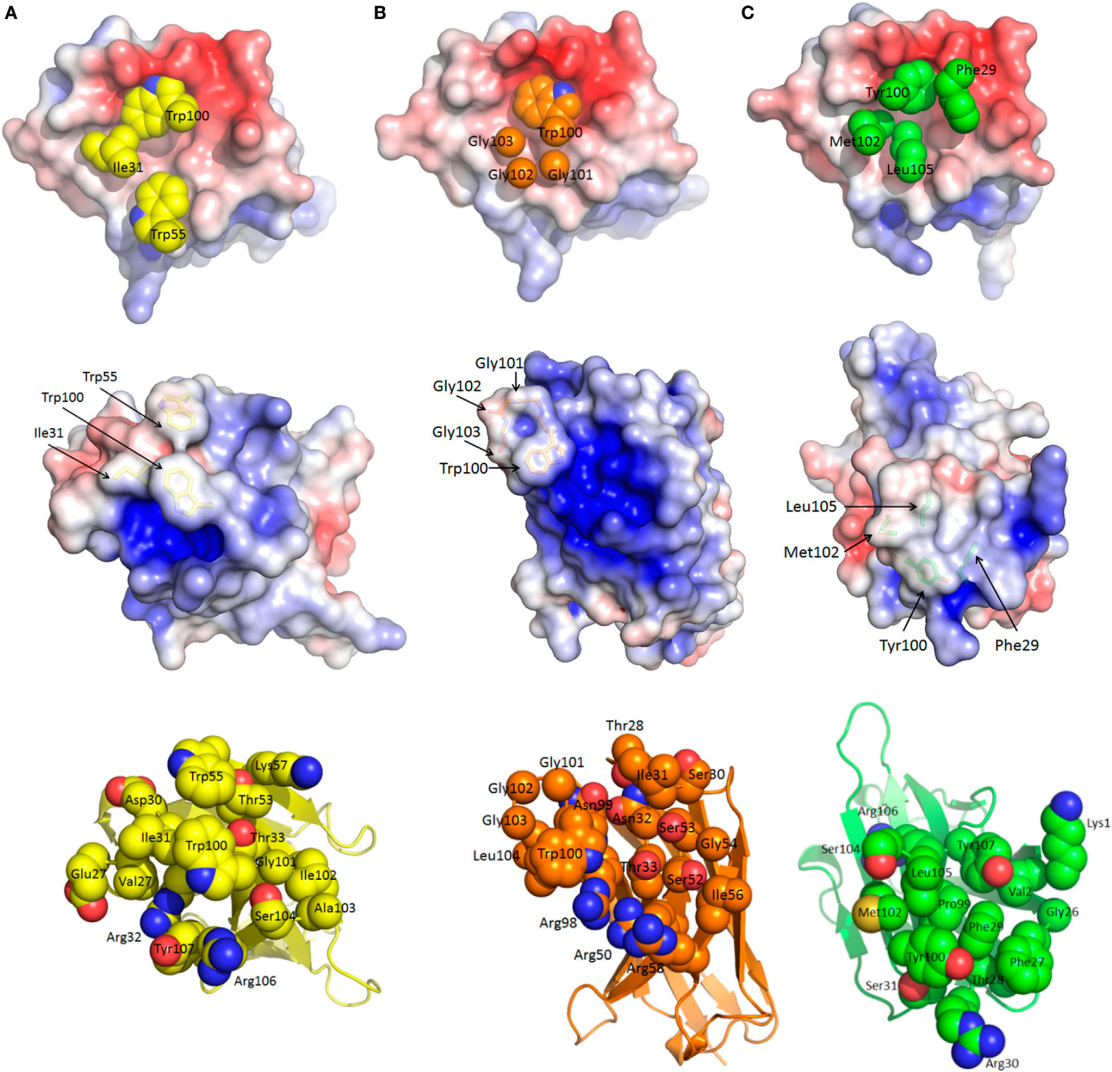
Common and unique features of sdAb–nucleoprotein (NP) engagements. For each sdAb A **(A)**, B **(B)**, and C **(C)**, the epitope is shown first (*top*) with the apical CDR3 aromatic group and other hydrophobic side chains occupying the basin. *Middle* images reveal the sdAb undocked, rotated 180° toward the reader and displayed with electrostatic surface potential [−5 (red) to +5 (blue) K_b_T/e_c_] to reveal the epitope’s eye view of the paratope. The convex hydrophobic networks of the basin filling residues are evident with side chains shown as sticks and annotated. The recessed differential positive charges that engage the negatively charged basin overlook become apparent especially for sdAb B. *Lower* images show the same view of each sdAb as the middle view with each residue predicted to be involved in antigen engagement shown as space-filled representations and labeled. The latter view serves to decode the electrostatic surface and more clearly shows the unique approach angles as the frameworks become more evident to the rear of each sdAb.

Figures 3A–C summarize the shape and charge complementarity between NP epitope and sdAb A, B, and C, respectively. Top is the sdAb’s eye view of the NP C-terminus as electrostatic surface potential occupied by key hydrophobic paratope residues. The pivotal CDR3 aromatic residue of each sdAb appears nestled toward helix 2 Asp679 and close to Leu676 plus Val691 borne on helix 1 and Met683 on the turn between helices 1 and 2. sdAb A and B dispose Trp100 almost at right angles to each other while sdAb C employs Tyr100. Since Tyr100 of sdAb C is slightly more toward the open end of the basin, this allows Phe29 to engage Asp679, Met683, and Val691 toward the closed end. Secondary hydrophobic areas in the basin formed by Leu663, Tyr667, Leu695, and again Val691 afford suitable accommodation to Ile31, Trp55 of sdAb A, Gly101-103 of sdAb B, and Met102 plus Leu105 of sdAb C.

The electrostatic surface potentials of the undocked sdAb flipped 180° from binding NP (middle of Figures 3A–C) give an epitope’s eye view of each paratope clearly showing the prominence of the hydrophobic CDR residues that engage the basin. Both sdAb A and C appear to exhibit the classical convex paratope, with relatively large contiguous regions of hydrophobicity, while sdAb B appears less pronounced. Differences in the number and distribution of positively charged paratope residues engaging the negatively charged basin overlook are apparent, and salt bridge and hydrogen bonding potential were revealed by PDBePISA analysis (44). While only Arg106 of sdAb A salt bridges Asp679, sdAb B employs Arg98, Arg50, and Arg58 to engage Glu675 plus Asp679, Asp679, and Asp682, respectively. While sdAb C also shares the Asp679 salt bridge route (with Arg30), this antibody is highly unusual in employing Lys1 of FR1 to engage Asp686 in a second salt bridge. Perhaps this alternative approach to binding may partially compensate for not employing CDR2, a feature only shared with one other sdAb to date (45). Amino acids Glu675, Asp679, and Glu687 are also involved in hydrogen bonding all three sdAb with Asp682 additionally H-bonding sdAb B. Hydrogen bonding potential is also predicted for Asn669 to sdAb A, Ser684 and Ala678 to sdAb B, His690 and Ser672 to sdAb A and C, and finally Tyr667 to sdAb B and C.

The lower panels of Figures 3A–C show space-filling representations of all predicted paratope residues giving an indication of the potential breadth of interactions. Here, the different approach angles shown in Figure 1A are also reflected in the differential visibility of conserved framework areas. The distribution of paratope residues of sdAb C appears more concentrated than either sdAb A and B, resembling an oval focusing on the basin interior. Together with the absence of additional helix crosslinking mediated by CDR2 and Tyr100 as shown in Figure S10 in Supplementary Material, these deficits may help explain the conformational sensitivity of sdAb C. An additional view of the three sdAb docking is shown in Figure S11 in Supplementary Material. The diverse potential for protein–protein interactions within the MARV C-terminus appears striking, being leveraged by all three sdAb in both unique and overlapping ways, while still preserving the rule of hydrophobic core and hydrophilic surrounds for the complex (46, 47).

Additional PDBePISA analysis of the crystal structures compares the antibody–antigen interfaces according to buried surface area, solvation free energy gain (Δ^i^G) from forming the interface, and the *P*-value of Δ^i^G which can be described as a value of interface specificity (a lower number <0.5 correlates with higher specificity). The buried surface area values are similar to 686, 653, and 663 Å^2^, respectively, for sdAb A, B, and C complexes. The interfaces have values for Δ^i^G and the *P*-value of −7.4 kcal/mol and 0.262, −4.5 kcal/mol and 0.375, and −8.5 kcal/mol and 0.118, respectively, for sdAb A, B, and C complexes. The values calculated for sdAb B and C complexes were averaged over the four complexes in each asymmetric unit, while values for the sdAb A complex were calculated for the single complex in its asymmetric unit.

### Complementarity Requires CDR Restructuring by All Three sdAbs

The shape complementarities (Sc) for the sdAb within the complexes were calculated using the CCP4 suite (48) and are 0.77, 0.52, and 0.67 for A, B, and C, respectively. Bearing in mind, Sc values for several immune antiviral Mab/Fab are in the 0.6–0.8 range (49, 50), the sdAb values are remarkably high for non-immune semi-synthetic sdAb from a single-pot library that have not undergone any affinity maturation. A non-immune antibody is only as good as its antigen and we are left with the sense that serendipity has offered up a remarkably attractive epitope for these sdAb to engage MARV NP. The lower Sc for the lower affinity sdAb B might be in part due to the presence of only one large hydrophobic group in the basin accompanied by three small Gly side chains, while sdAb A and C have two aromatic side chains and bulkier hydrophobic Ile and Leu residues, respectively.

**Figure 4:**
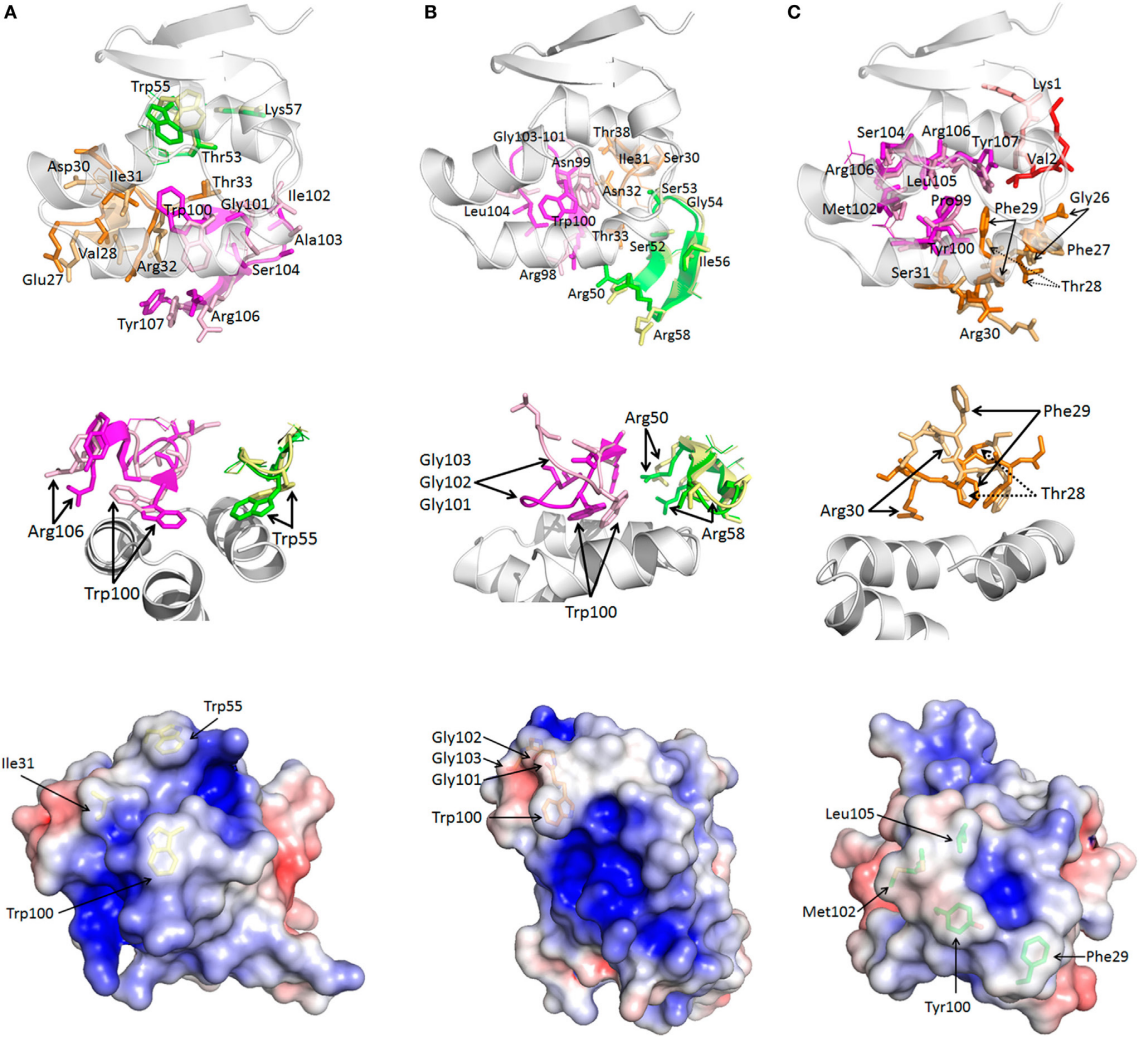
Comparison of bound and unbound sdAb reveals complementarity-determining region (CDR) restructuring. For each sdAb A **(A)**, B **(B)**, and C **(C)** (*top*) looking up from beneath the nucleoprotein (NP) epitope (white) at the bound/unbound CDR1 (orange/beige), CDR2 (green/yellow), CDR3 (magenta/pink), and FR1 (red/salmon for sdAb C) shows CDR loop rearrangements. Residues predicted to engage antigen are displayed in stick form and labeled while those that do not are shown as lines and unlabeled. *Middle* images show each interaction colored as above but aspects have been chosen to more clearly show the primary fits occurring with CDR1 removed for clarity for sdAb A and B, and only CDR1 shown for sdAb C. *Bottom* images present unbound sdAb with electrostatic surfaces and the basin occupying residues shown as sticks. Note that Glu1 and Val2 are not visible in sdAb A while Lys1 is not visible in sdAb C. Two isoforms of Met102 are visible in sdAb C, neither corresponding to the bound disposition. When comparing the unbound sdAb structures to the bound sdAb structures (Figure 3 middle), the hydrophobic side chains destined to occupy the NP basin appear more diffuse in sdAb A and sdAb C, while sdAb B has equivalent density yet has a more glancing side-on CDR3 disposition.

When free and bound sdAb are compared (Figure 4), it becomes clear that each antibody still undergoes substantial restructuring as a means to improve antigen recognition (51). sdAb A exhibits a 180° flip for Trp100 and Trp55, with Ile31 also needing adjustment to present a more tightly knit array of hydrophobic side chains evident in the bound electrostatic surface shown in Figure 3. sdAb B CDR3 extends and flattens when bound to enable Trp100, Gly101-103, and Ile104 better access to the basin interior. Arg58 and, to a lesser extent, Arg50 at the landing and take-off sites of CDR2 also shift to reach their salt-bridging partners on the basin overlook. sdAb C is unusual out of the three antibodies in that CDR3 appears to be a reasonable pre-existing fit already, with the majority of fitting occurring in CDR1. Here, the main chain undergoes an S curve reversal (i.e., S to ϩ) to move Phe29 toward the basin with an ~11 Å maximal repositioning to displace the neighboring Thr28 which shifts by ~6 Å. The final position of Phe29 is almost a supporting role to Tyr100, but it does have modest contacts of its own. Amino acids Arg30 of CDR 1 and Lys1 of FR1 also move to meet their respective salt bridge partners on the overlooks with both having ~9 Å shifts.

Comparison of free and bound forms of a highly unusual human broadly neutralizing Ab, capable of neutralizing all serotypes of influenza A, has recently been shown to exhibit dramatic CDR restructuring (52). The movements enable better accommodation of aromatic and hydrophobic residues within a hydrophobic groove of HA, with a key CDR3 Phe showing a ~5 Å shift. By virtue of having missing electron density in CDR3 of the free form, an anti-HIV gp120 immune llama sdAb capable of cross-clade neutralization may also employ restructuring to fit (53), though the bound form will be required to confirm this. It may well be that the potency of antibody repertoires for cryptic viral antigens not only relies on the total number of unique clones but also on the ability of the CDRs to accommodate such dramatic tertiary changes on transitioning from free and soluble forms to bound and potentially insoluble forms.

### Conservation of the sdAb Cryptotope

**Figure 5:**
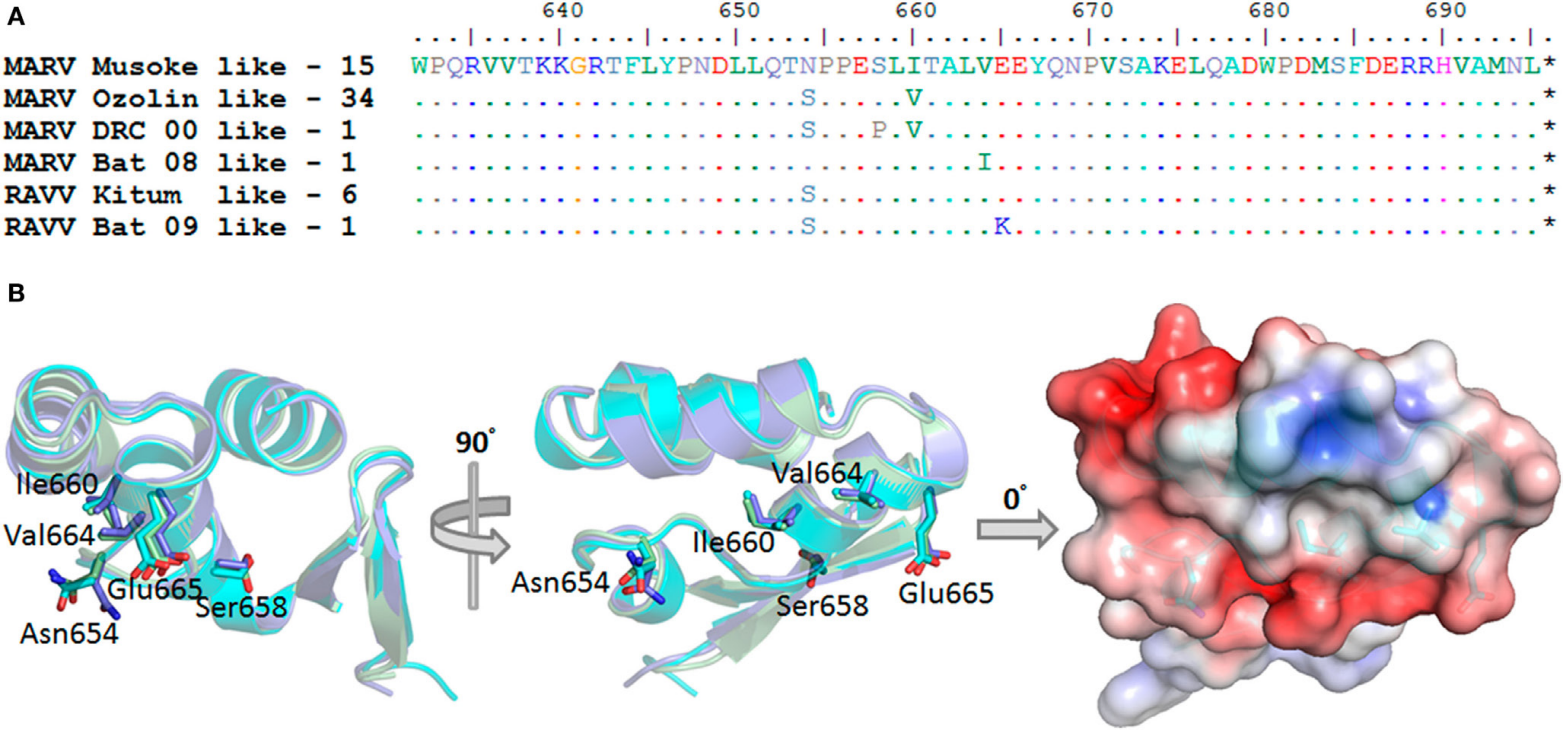
Natural evolution of the Marburg virus (MARV) nucleoprotein (NP) C-terminus is remote from the sdAb epitope. **(A)** Summary of the frequency with which human and bat MARV and Ravn virus (RAVV) NP genes vary within the C-terminal region under study. **(B)** The C-terminal domains and side chains are displayed end or side on as if sdAb were binding the epitope from above as in Figure 1A. The amino acids prone to change are identified as sticks and labeled to reveal side chains that do not overlap the sdAb epitope, lying beneath and to one side of the domain. The electrostatic representation of the domain derived from the sdAb C complex is rendered on the right.

Alignment of MARV NP amino acid sequences from humans and bats since 1967 derived from the Los Alamos Filovirus database https://www.hfv.lanl.gov (54) revealed positions prone to mutation within the C-terminus summarized in Figure 5A. Using Musoke (1980, *n* = 1) as our parental baseline the Leiden (2008, *n* = 1), Popp/Ci67 (1967, *n* = 2), Angola (2005, *n* = 8), Ugandan (2012, *n* = 2), and one Ugandan bat strain (2009, *n* = 1) are all homologous, highlighting conservation across almost 50 years of evolution. One Uganda bat sequence has Val664Ile (2008, *n* = 1). Ozolin (1975, *n* = 1) has Asn654Ser and Ile660Val which also occur together in many human isolates from DRC (1999/2000, *n* = 27) and several Ugandan bat sequences (2007, *n* = 2, 2008, *n* = 1 and 2009, *n* = 3). Within the DRC outbreak, one sequence had Ser658Pro in addition to Asn654Ser and Ile660Val (2000, *n* = 1). Human RAVV sequences from Kenya, DRC, and Uganda differ from Musoke in having Asn654Ser (Kitum Cave 1987, *n* = 1; DRC 1999, *n* = 1; Uganda 2007, *n* = 1) which also occurs in Uganda bat sequences (2007, *n* = 2; 2008, *n* = 1) with one additionally having Glu665Lys (2009, *n* = 1). When residues prone to drifting are mapped on to the C-terminal structure, all reside on helix 3 or just beyond it with their side chains disposed away from the epitope (Figure 5B). The relaxed contact mapping analysis (Figure S10 in Supplementary Material) also failed to predict these amino acids as involved in engaging the sdAb. We had previously shown that all four sdAb showed equivalent responses in sandwich capture of Triton-lysed RAVV when compared with Musoke and Angola viruses [Figure 1 of Ref. (21)], showing experimentally that at least Asn654Ser alone did not appear to impact binding. Furthermore, any subtle impacts on affinity due to these mutations are likely to be overcome by avidity effects within the sandwich assay format as indicated by our lower EC_50_ values derived from polyvalent versus monovalent binding assays.

The only other known anti-NP MARV antibody we are aware of that has been mapped to the MARV C-terminus is a mouse Mab shown by deletion mutagenesis to require amino acids 643–695 (55). Without structural information, it is difficult to assess exactly where and how this antibody binds, and whether it is likely to be impacted by MARV variation or not. It would be of great interest to compare and contrast the footprints of our sdAb with the conventional IgG, to determine if they share similar approaches to binding the NP C-terminus or not.

### Similarities and Differences between the MARV and EBOV C-Termini

**Figure 6:**
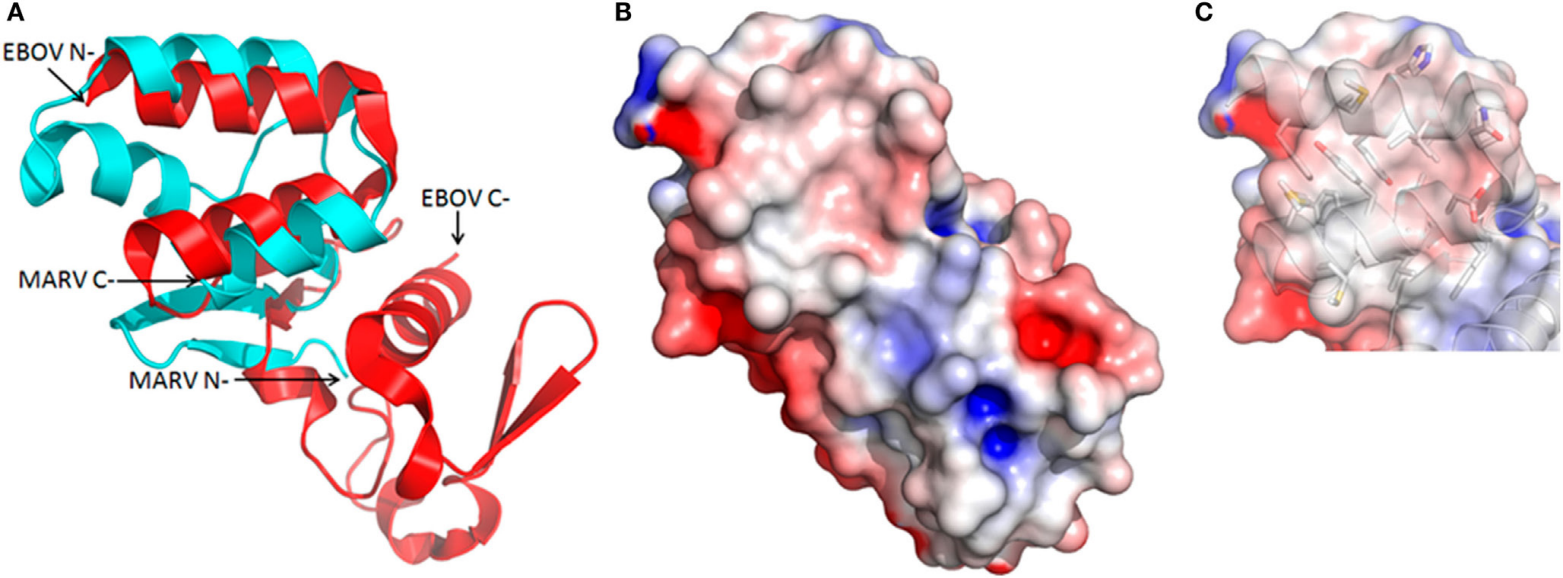
Structural but not positional homology between Marburg virus (MARV) and EBOV nucleoprotein (NP) C-termini. **(A)** Cartoon overlay of the *Ebolavirus* (EBOV) (red) and MARV (cyan) C-termini as deduced from a Dali homology search reveals the two alpha helices forming the upper V-shelf are somewhat conserved. Note that while the V-shelf is at the extreme C-terminus of the MARV NP it is internal to the EBOV NP C-terminus. **(B)** Electrostatic surface potential reveals a much shallower and compact basin for EBOV. **(C)** Reduced basin width in EBOV is primarily due to Phe648 and Tyr652 from one helix stacking with Tyr667 from the other to form a wall-like structure that fills in the cavity as opposed to shorter side chains lining the MARV basin (cf. Figure 2D).

That our sdAb epitope appears resistant to natural evolutionary variation suggests a critical function in viral replication such as interfacing with host proteins or other viral proteins. Such protein–protein interfaces are generally more conserved than non-interface surfaces (56) since mutations in one surface may require compensatory mutations in the other and will be less likely to occur. If the interface becomes part of the virion, as would occur if it was between two viral structural proteins, it will only be exposed upon virion dissociation (57). A 3D structural homology search using the Dali server (58) identified the C-terminal structures of Zaire (59), Bundibugyo, and Tai Forest (35) viruses as homologous to our MARV domain *via* the two last alpha helices. Perhaps surprisingly, overlaying the MARV and EBOV (Zaire) structures (Figure 6A) reveals that the EBOV motif is not at the C-terminus but 66 residues upstream indicating there is plasticity in where the motif needs to be in order to function. Secondary structure prediction using JPred (60) was unable to identify the preceding residues as prone to alpha helix formation, suggesting that in EBOV the basin may well rely on just the V-shelf helices without a third helix forming the basin floor. Indeed, the EBOV basin is comparatively shallow (Figure 6B) and smaller than MARV with a wall of stacked aromatic side chains between the helices occupying potential inter-helix space (Figure 6C). The more open end of the EBOV shallow basin appears to be across the axis of one of the helices between Ala664 and Val665 which create a dip rather than a route out over the Tyr667 of MARV (cf. Figure 2D). The basin overlooks of EBOV are not highly negatively charged with only Asp663 appearing to share a similar position to the Glu687 of MARV. The differences between MARV and EBOV motifs imply that if they do have similar roles in protein–protein interactions they may use alternative approaches to engage their particular partner protein(s). The differences also explain why our anti-MARV sdAb do not cross-react among the EBOV genus [Figure 1 of Ref. (21)] since the shape and charge complementarities required for sdAb binding are absent.

## Discussion

To our knowledge, our study represents the first high-resolution structural study of an antibody binding a filoviral NP. As such, the information can guide us through structure based design to improve the performance of the sdAb by focused *in vitro* evolution or educated mutagenesis. NP is an important biomarker for Marburg hemorrhagic fever, and high-end antibodies to conserved epitopes that may push the limits of detection toward nucleic acid test levels would be a significant step forward for point-of-care tests. The innate thermal stability of the sdAb format may make the resulting assays more suitable for resource poor environments where cold-chains are lacking. A mandate for conservation of the sdAb epitope, to play a vital role in viral replication, bodes well for its long-term utility in enabling sdAb to recognize MARV and RAVV strains yet to emerge.

While crystal structures of constructs bearing amino acids 19–370 (61) and 552–579 (25) of MARV NP have been resolved, the remaining C-terminal region has proved more challenging, existing as a molten globule (35). Herein, by engaging the MARV C-terminal region with sdAb we overcame this roadblock. While two of the sdAb performed well as crystallization chaperones, the third (sdAb C) required much optimization for success, suggesting the approach is still somewhat empirical. However, since we were unable to generate any crystals of NP600, NP632, or the fixed arm maltose binding fusion protein equivalents, trans-sdAb rather than cis-mbp chaperoning appeared essential for success in this case. While we cannot rule out contributions to crystal packing afforded by the hydrophilic surface of the sdAb, it is more likely their role was to reduce conformational heterogeneity (62) of the MARV C-terminus to allow crystals to form. We do not know the precise choreography that occurs when transitioning between free and bound sdAb, only the end-points. It could be that the sdAb architectures were encouraged to form a more focused hydrophobic apical core, around which the basin could form from the molten state and the overlooks could be subsequently crosslinked to “fix” the MARV C-terminus. Alternatively, the molten state may transition through a folded C-terminal structure, which was then selectively extracted by the sdAb over time. Since all of these recombinant fragments are highly productive and relatively small, it should be possible to further explore the contributions of induced fitting and conformational selection using biophysical techniques.

**Figure 7:**
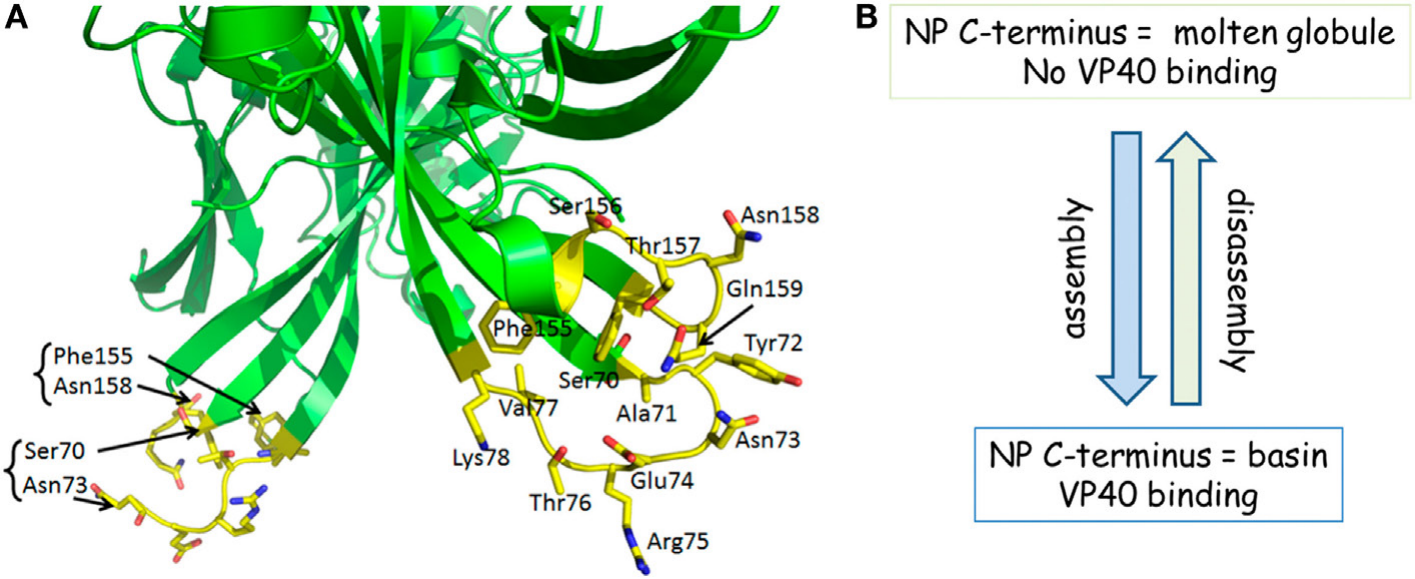
**(A)** Marburg virus (MARV) VP40 appears to have loops that resemble complementarity-determining regions (CDRs). Loops in the region distal to the membrane binding patches (out of view) of the MARV VP40 dimer show a striking similarity to antibody CDRs, stemming from a scaffold crudely resembling frameworks. While one loop is visible in the crystal structure, the other is not which implies a flexibility that might be employed for restructuring. **(B)** A summary of our working hypothesis that the transition from disorder to order and *vice versa* within the nucleoprotein (NP) C-terminus is a molecular switch for virus assembly and disassembly by being able to host or release VP40.

It is tempting to speculate that like EBOV (63), the MARV C-terminus engages VP40 matrix protein for virus particle assembly, resulting in a layer of matrix between the polyvalent NP of the ribonucleocapsid and the viral membrane (22, 32). If we consider portions of the sdAb paratopes as mimics of VP40, much the same as some anti-influenza A virus broadly neutralizing antibodies can mimic portions of the influenza virus A HA receptor (64, 65), the loops revealed in the crystal structure of the MARV VP40 dimer (66) could potentially play this role (Figure 7A). The loops appear borne on scaffold-like structures that uncannily resemble CDRs borne on frameworks of antibodies. While one set of loops is visible in MARV VP40, there is missing electron density in the other set (Ser156, Thr157 and Ala71, Tyr72) indicating enough flexibility to undergo restructuring if required. Though it is impossible to draw definitive conclusions based on the structure of the complete MARV VP40 loop that is visible since it is involved in crystal packing, the occurrence of Phe, Thr, Tyr, and Arg residues may indicate involvement in protein–protein interaction since these residues are all highly favored at interfaces (47, 56). The fit between VP40 and NP need not be perfect nor high affinity since the “unusual, flexible Velcro-like” interaction (22) when polyvalent nucleocapsids laterally meet VP40 lattices for assembly at the membrane (67) could capitalize on avidity. The NP C-terminus is regularly displayed on the outer face of the nucleocapsid several thousand times and would be an ideal candidate to be proximal to the loop regions of VP40. Furthermore, during disassembly following virus entry and fusion, a weak interaction between VP40 and NP would be preferable for rapid dissociation to enable the nucleocapsid to be delivered to the cytoplasm efficiently. The high prediction of disorder at the C-terminus of MARV (68) combined with prior observations of the molten globule with three alpha helices present (35) suggests that our current crystal structure may represent the more orderly end of a dynamic molecular switch for virus assembly and disassembly (Figure 7B).

## Materials and Methods

### General Cloning

Recombinant DNA methods were according to established procedures and employed commercially available reagents; Phusion High-Fidelity DNA Polymerase (Thermo Fisher, Waltham, MA, USA); restriction enzymes and β-agarase (New England BioLabs, Beverly, MA, USA); T4 DNA ligase, CIP and T4 PNK (Roche, Nutley, NJ, USA); GTG low melting temperature agarose for in gel cloning (Lonza, Walkersville, MD, USA); oligonucleotides (Integrated DNA Technologies, Coralville, IA, USA); cloned synthetic DNA (Genscript, Piscataway, NJ, USA). Assemblies involving cloning and PCR amplification were sequenced through the inserts and junctions to verify the desired construct. Cloning was typically carried out in XL1-Blue cells unless otherwise stated. Parental sdAb genes employed in this work were anti-MARV NP–sdAb A, B, C, and D with GenBank accession numbers MF780583, MF780584, MF780585, and MF780586, respectively. Full details of cloning, oligonucleotides, maps, and sequences of resulting constructs are available on request.

### Expression and Purification of sdAb from *E. coli* for ELISA

For the NP sandwich ELISA freshly made soluble anti-MARV NP sdAb A, B, C, and D proteins derived from *lac* promoter and *pel*B signal-based periplasmic secretion vector pecan22 (21) were expressed and purified from 500 mL scale cultures in *E. coli* Tuner + pRARE. Clones were grown in 50 mL starter cultures of terrific broth (TB) plus 2% glucose at 30°C overnight with ampicillin (200 μg mL^−1^) and chloramphenicol (30 μg mL^−1^) in 250 mL Bellco baffled flasks. Saturated cultures were transferred to 450 mL of fresh TB without glucose and shaken for 3 h at 25°C in 2,500 mL Bellco baffled flasks. Expression was induced by addition of IPTG to 1 mM for 3 h at 25°C, the cells pelleted (typical wet weights of 8–9 g) and osmotically shocked (69) by resuspension in 14 mL ice-cold 0.75 M sucrose in 100 mM Tris–HCl pH 7.5, addition of 1.4 mL of 1 mg mL^−1^ hen egg lysozyme (Sigma), followed by drop-wise addition of 28 mL of 1 mM EDTA pH 7.5 and swirling on ice for 15 min. 2 mL of 0.5 M MgCl_2_ was added, swirling continued for 15 min and cells pelleted. The 45 mL supernatant (osmotic shockate) was mixed with 5 mL of 10× IMAC (IMAC buffer—0.2 M Na_2_HPO_4_, 5 M NaCl, 0.2 M imidazole, 1% Tween-20, pH 7.5), followed by 0.5 mL of High-Performance Ni Sepharose (GE Healthcare) and the suspension gently mixed on ice for 1 h. Resin was pelleted at 3,000 rpm for 5 min (Beckman Allegra 6R swing out rotor) and washed twice with 50 mL of 1× IMAC solution before elution with 2 mL 0.5 M UV-grade imidazole in 1× IMAC buffer, pH 7.4. Proteins were concentrated in Amicon 10 kDa ultrafiltration devices (Millipore, Billerica, MA, USA) to 200 µL for separation by gel filtration on a Superdex 200 10/300 or Increase 10/300 GL column (GE Healthcare, Pittsburgh, PA, USA) operating in PBS. Proteins were quantified by UV adsorption and analyzed by SDS-PAGE and Coomassie blue staining for impurities.

### Expression and Purification of Recombinant NP from Human Embryonic Kidney (HEK) 293T Cells

Human embryonic kidney 293T cells (ATCC, Manassas, VA, USA) were grown in Dulbecco’s modified Eagle’s medium (DMEM) with 4.5 g L^−1^ glucose, l-glutamine, sodium pyruvate (Corning cellgro), 5% fetal bovine serum (Corning, NY, USA), and penicillin/streptomycin (complete medium) at 37°C and 10% CO_2_ with humidity. Cells were seeded in sixteen 10 cm diameter dishes at 5e+6 cells per dish in 25 mL of complete medium 16–18 h prior to transfection. The backbone of pcDNASfi (27) was modified by deletion of three internal *Nco*I sites using Quick change mutagenesis (Stratagene) and synthetic DNA encoding a portion of the hCMV promoter and intron A was mobilized from pUC57 CMV-INTA *via Sna*BI and *Nhe*I to replace the resident 5′-ntr to create puma2. Previously described human codon-optimized genes residing in pcDNASfi encoding Marburg Musoke (MARV), Ebola Zaire Kikwit (EBOV), or Ebola Bundibugyo NP were back inserted to puma2 *via Sfi*I. Qiagen miniprep DNA (105 µL at 100 ng μL^−1^) and 41 µL linear polyethylenimine (1 μg μL^−1^, pH 7.0) were combined and equilibrated for 20 min at room temperature in 2.5 mL serum-free DMEM prior to being carefully added to the medium. Cells were collected 48 h post transfection by trypsinization in 4 mL trypsin–EDTA solution (Sigma, St. Louis, MO, USA) with 2-plates worth of cells combined into 50 mL Falcon tubes and topped up to 50 mL with phosphate-buffered saline (PBS). Cells were pelleted at 1,000 rpm for 5 min (Beckman Allegra 6R swing out rotor) washed once with PBS and repelleted. The cells were lysed in 4 mL of ice-cold hypotonic buffer consisting of 20 mM HEPES pH 7.5, 5 mM KCl, 1.5 mM MgCl_2_, 1 mM DTT, 1 tablet of cOmplete™ EDTA-free protease inhibitor cocktail (Roche) per 50 mL. DNA was sheared by passing through a 30-G needle several times on ice. Samples were microfuged in 2 mL tubes at 6,000 rpm for 10 min at 4°C (5415D microcentrifuge, Eppendorf, Hauppauge, NY, USA) and the supernatants transferred to fresh tubes and re-centrifuged at 13,000 rpm for 10 min. Clarified samples were pooled and concentrated in two 15 mL 100 kDa cut-off Amicon centrifugal filters at 3,500 rpm (Beckman Allegra 6R, swing out rotor, room temperature) until the volume was approximately 800 µL. Samples were clarified by microcentrifugation at high speed for 5 min immediately before loading 400 µL on to CsCl gradients (40–25%, 5% steps in TNE—10 mM Tris–HCl pH 7.4, 150 mM NaCl, 1 mM EDTA). Gradients were centrifuged at 25,000 rpm (Beckman SW41Ti) for 18 h at 20°C. The NP bands were collected by side-puncture with an 18-G needle, samples combined and dialyzed in 10 kDa cut-off Slide-A-Lyzer cassettes (ThermoFisher Scientific) against PBS at 4°C. Samples were quantified by micro-BCA assay and analyzed by SDS-PAGE and silver stain. Samples were made to 15% glycerol, aliquoted and flash frozen in liquid nitrogen and stored at −80°C.

### NP Sandwich ELISA

sdAbs were used to coat duplicate Costar white high binding ELISA plate wells at 100 µL of 100 nM in PBS overnight at 4°C. Plates were rinsed with PBS and wells blocked with 2% non-fat dried milk (Carnation, MPBS) to brimming for 1 h at room temperature. Purified NP in MPBS was serially diluted over the sdAb and incubated for 5 min shaking. Plates were washed three times with PBS + 0.1% Tween-20 and two times with PBS. Phage displayed versions of the sdAb derived from pecan21 were used from the original stocks that had been stored at −80°C since 2007 and 1 µL used per well in 100 µL of MPBS for 5 min shaking. Plates were washed as before and 100 µL of 1/2,500 dilution of anti-M13KO7–HRP conjugate (GE Healthcare) in MPBS applied to each well and left for 5 min with shaking. Following washing, signals were developed with SuperSignal ELISA Pico chemiluminescent substrate (Thermo-Fisher) with 2 s integration using a luminometer (Turner Biosystems) and the duplicates averaged. The assay was performed two more times to create a graph representing the average of the three plots with maximum and minimum bars representing the SD. The negative was not a full titration but the maximum concentration of recombinant Bundibugyo NP.

### Aromatic Residue Knockout Analysis

Quick change site-directed mutagenesis was employed to mutate the CDR3 aromatic residue of sdAb A, B, and C to Ala and expressed in pecan126 which encodes a BAP sequence downstream of the sdAb (26). Proteins were expressed in HBV88 as for Tuner + pRARE and purified as above. 100 µL of 1 μg mL^−1^ purified MARV or EBOV NP in PBS was used to coat duplicate ELISA plate wells overnight at 4°C. Following rinsing and blocking as above, sdAb proteins in MPBS were titrated over the NP and left for 1 h static. Following washing, 100 µL of 1/10,000 anti-His_6_-HRP conjugate (Sigma) in MPBS was applied for 1 h. Following washing, the plates were developed and duplicate wells averaged. The ELISA was repeated once and curves represent the average of the two plots with bars representing SDs.

### Gluc-Based EC_50_ Determination

The sequence encoding an *E. coli* codon-optimized Gaussia luciferase (gluc) gene within pUC19 from the NanoLight™ Technology website (Pinetop, AZ, USA) was used as the basis for designing overlapping oligonucleotides encoding the open reading frame plus a His_6_ sequence flanked by unique *Nco*I and *Hin*dIII compatible overlaps. Following kinasing, the oligonucleotides were heated and slowly cooled in Taq DNA ligase buffer, enzyme added and ligated to gel purified pecan22 from which a resident sdAb gene had been removed with *Nco*I and *Hin*dIII. A faithful clone was used to confirm active gluc enzyme could be expressed and purified at 500 mL scale as above and then the gene was re-engineered to enable insertion of recombinant antibody fragments. Hingeless sdAb A-D genes from pecan73 were subsequently inserted *via Nco*I and *Not*I to generate the pecan35 sdAb–gluc gene fusions. The resulting glucibodies were expressed and purified as for sdAb above within Tuner + pRARE.

Recombinant NP of either MARV or negative control Bundibugyo Ebola in 100 µL of PBS at 1 μg mL^−1^ were used to coat duplicate wells of ELISA plates at 4°C overnight. Plates were washed three times with PBS and each well blocked to brimming with MPBS for 1 h. Wells were then probed with 100 µL of the gluc control or glucibody dilutions in MPBS for 1 h static. Probe was removed and plates washed three times with PBS containing 0.1% Tween-20 (PBST) and two times with PBS. Signals were developed with injection of coelenterazine (NanoLight™ Technology) in lucky buffer (10 mM Tris, 1 mM EDTA, 500 mM NaCl, pH 7.4) and collected using the luminometer with a 2 s integration. Duplicate wells of each dilution were averaged and the Bundibugyo NP signals subtracted from the MARV NP signals. The titrations were repeated twice with the final plots representing the mean of three experiments and the error bars representing ± SD. The EC_50_ *y*-value was calculated for each curve using the equation [RLU_min_ + (RLU_max_ − RLU_min_)/2]. The corresponding *x* values were calculated using one observed point greater and one less than the *y* EC_50_ using the trend function in Excel and the three values averaged and presented ± SD. Statistical significance was determined using a paired two-sample Student’s *t*-test with an alpha value of 0.05 within the Excel data analysis toolpak.

The *mal*E gene from XL1-Blue was amplified to encode a modified N-terminus of MetLysIleHis_6_ (70) and a C-terminal fixed arm of Ala_3_ encoded by a *Not*I restriction site (71) and inserted into pE (see below) *via Nde*I and *Hin*dIII. An oligonucleotide bridge encoding Ala_3_GlySer was then inserted between *No*tI and *Hin*dIII sites to create a control maltose-binding protein (mbp) gene, while NP600 and NP632 were amplified and inserted between the *Not*I and *Hin*dIII sites to create the mbp-NP600 and mbp-NP632 fusion protein expression vectors. Proteins were expressed, purified, quantified, and analyzed by SDS-PAGE and then substituted for recombinant NP as immobilized antigen in the glucibody EC_50_ determination above. Signals on the mbp control protein were subtracted from the mbp-NP600 and mbp-NP632 signals and the experiments repeated three times to generate plots representing the means with error bars representing ± SD. Statistical significance was determined using a paired two-sample Student’s *t*-test with an alpha value of 0.05 within the Excel data analysis toolpak.

### NP Deletion Mutagenesis

Phagemid pecan42, a *tac* promoter-based vector harboring the MARV Musoke NP gene with a C-terminal His_6_ tag (21) was first used as a template for introducing an N-terminal FLAG tag by splice-overlap extension (SOE) PCR. Stepwise deletions of 100 amino acids (the C-terminal region was 95 amino acids) from the authentic NP initiation codon were then made using SOE-PCR. Clones were mobilized to Tuner + pRARE and 20 mL expression cultures used to generate lysates from 20 OD units in 2 mL tubes using a Mini-beadbeater 16 (Biospec Products). Lysates (10 µL) were Western blotted to Immobilon P (Millipore) for probing with anti-FLAG M1-HRP conjugate (Sigma), anti-His_6_-HRP (Sigma) or the hyperactive AP fusions of each sdAb from pecan16 described previously (21) at 100 nM in MTBS (where Tris–HCl replaces phosphate buffer). Signals were developed with Lumi-Phos WB (Thermo-Fisher) sufficiently for each clone to reveal as much signal as possible without blowout.

### Production of NP600 for Crystallization

Phagemid pE is a T7 promoter-based vector assembled from the high copy number backbone of pecan but bearing a T7 cassette assembled from overlapping oligonucleotides to enable high yield of DNA from mini-preps to afford facile sequencing and manipulation and high gene dosage for expression. The perfectly symmetrical *lac* operator (72) ensures tight regulation within expression hosts like BL21 (DE3) despite the high copy number. The MARV Musoke NP C-terminus was amplified from pecan42 MARV NP and inserted into pE such that a MetGlyHis_6_GlyGlyGlySer sequence preceded the NP sequence. 50 mL overnight starter cultures with BL21(DE3) + pRARE in TB with 2% glucose, ampicillin at 200 μg mL^−1^ and chloramphenicol at 30 μg mL^−1^ were grown at 30°C until saturation. Cultures were poured into 450 mL glucose-free TB, grown with vigorous aeration in Bellco baffled flasks for 3 h at 25°C and induced for 3 h with 0.1 mM IPTG. Cultures were centrifuged and the pellets drained of excess media and stored at −80°C until ready for beadbeating. Once thawed, the pellets were resuspended in 40 mL 1× IMAC plus a complete protease inhibitor tablet (Roche) and added to a 50 mL chamber filled halfway with 0.1 mm glass beads. The chamber was topped off with 1× IMAC buffer to remove any air bubbles and the cell/bead mixture was blended on ice within a 4°C fridge for a total of 12 min with 2 min on and 2 min cooling on ice in between. Once contents settled, the cell debris was transferred to a 50 mL conical tube and centrifuged at 3,000 rpm for 15 min at 4°C (Beckman Allegra 6R, swing out). The supernatant was decanted into a new 50 mL tube and centrifuged at 9,500 rpm for 15 min at 4°C (Sorvall RC 6+, F13 FiberLite rotor). The supernatant was filtered through a 32 mm diameter 0.8/0.2 μm filter (Pall) and applied to a 5 mL HisTrapHP column equilibrated in 1× IMAC. Protein was eluted with a 0–500 mM imidazole gradient in 1× IMAC buffer. The fractions were pooled and dialyzed into 20 mM Tris–HCl pH 7.4, 5% glycerol and loaded onto a column (20 mL bed volume) of High-Performance Q-Sepharose resin (GE Healthcare) equilibrated in 20 mM Tris–HCl pH 7.4. The protein was eluted with a 0–500 mM sodium chloride gradient, pooled, and concentrated to 2 mL. The sample was further purified on a Superdex 75 16/60 column in 10 mM Tris pH 7.4, 150 mM NaCl. Protein was quantified by UV adsorption and analyzed by SDS-PAGE to access purity. For crystallography, preparations were diluted to 12 mg mL^−1^, aliquoted and stored at −80°C.

Western blotting of tenfold dilutions of NP600 employed 100 nM of the sdAb–AP fusions in MTBS with each probed membrane subsequently aligned side-by-side for simultaneous development to ensure accurate comparison across the sdAb clones.

### nluc-Based EC_50_ Determination

A pE variant (pENCO1) was first engineered where the ATG start codon was within an *Nco*I site rather than an *Nde*I to allow genes coming from *pel*B leader constructs to be shuttled conveniently over. A synthetic gene encoding nluc based on the Promega website (Madison, WI, USA) with and without the single Cys had been explored for secretion capacity in pecan73 (26) (a *tac* promoter *pel*B leader vector) as a C-terminally His_6_-tagged motif and found very lacking. The nluc Cys minus gene was therefore mobilized from the periplasmic to the cytosolic system to create pENCO9 for control protein production. MARV Musoke NP600 and NP632 were separately fused to nluc using SOE-PCR such that the gene fusions sandwiched the His_6_ tag between the nluc and NP domains. Proteins were expressed, purified, and quantified as for NP600 except that the dramatic solubility enhancement afforded by the nluc fusions obviated the need for ion exchange. ELISA plates were coated overnight at 4°C with 100 µL of 1 μg mL^−1^ of neutravidin in PBS. Plates were washed three times with PBS and then blocked by filling to brimming with Bioplex buffer (2% bovine serum albumin, 0.05% Tween-20 in PBS) for 1 h. 100 µL of 100 nM sdAb as a BAP fusion purified from pecan126 as described above was applied to the well in Bioplex buffer for 1 h. Wells were washed to brimming three times with PBST and two times with PBS. MPBS was added to the well to brimming for 1 h to further block the sdAb and then dilutions of nluc, nluc-NP600, or nluc-NP632 in MPBS were added to duplicate wells for 1 h. Following washing the same substrate and buffer as used for gluc was added to wells and signals captured as above. The experiment was repeated two more times and curves are the plots of three mean RLU of nluc-NP600 or nluc-NP632 minus the corresponding mean of the nluc alone with error bars representing SD. The EC_50_ values were determined from individual curves as above and statistical significance determined likewise.

### Production of sdAb for Crystallization

Genes encoding sdAb A, B, and C were first mobilized to pecan73 using PCR to delete the flexible llama Ig hinges and fuse the His_6_ tag closer to FR4. Expressions and harvesting at 500 mL scale were initiated as above and the shockate was made to 100 mM NaCl, 10 mM imidazole, and 5% glucose and frozen at −80°C prior to purification. sdAb was captured using a 5 mL HiTrap sepharose column (GE Healthcare) charged with nickel and equilibrated with TIGS buffer (100 mM Tris–HCl pH 7.4, 100 mM NaCl, 10 mM imidazole, and 5% glycerol). Bound protein was washed with three column volumes of TIGS buffer and eluted with a 10–270 mM imidazole gradient over 18 column volumes, pooled and dialyzed into 50 mM sodium phosphate, pH 7.0 with 5% glycerol. The protein was further purified on a HiLoad 26/600 SP Sepharose column (GE Healthcare). Bound protein was eluted with a 0–500 mM sodium chloride gradient, pooled and concentrated to 1 mL *via* Centricon ultraconcentration. Final purification of the sdAb A and B samples were carried out with a HighLoad 16/60 Superdex 75 prep grade column (GE Healthcare) equilibrated in 10 mM Tris–HCl pH 7.4 while sdAb C required additional 150 mM NaCl to not precipitate. Complexes of sdAb A and B with NP600 were obtained by overnight equilibration of 1:1 mixtures.

### Bait Prey Strategy to Generate sdAb C/NP632 Complex

Splice-overlap extension PCR was used to re-amplify the sdAb C gene from pecan73 to delete an internal *Nco*I site and terminate the ORF immediately after FR4 with no His_6_ tag. The product was back inserted into pecan73 *via Nco*I and *Hin*dIII to create pecan219 sdAb C. The first 31 amino acids of the pE-NP600 construct were deleted by PCR and back cloning to create pE-NP632 which was used to drive expression of NP632 as for NP600 as above. Culture volumes (2 L) yielding approximately two wet weight pellets of 28 g were bead beated and each partially purified on the 5 mL HiTrap IMAC column and gradient eluted. The peak fractions were combined and applied to the Q-Sepharose column as before and then combined with osmotic shockate derived from 4 × 500 mL pecan219 sdAb C cultures made to 1× TIGS and the mixtures stirred at 4°C overnight. The complex was batch IMAC purified and eluted as for sdAb, and purified on the S75 16/600 column in 10 mM Tris pH 7.5, 150 mM NaCl. The final sample was concentrated to 2 mL, quantified by micro-BCA assay (12.8 mg mL^−1^) and evaluated for purity by SDS-PAGE.

### Crystallization, Structure Determination, and Refinement

Automated screening for crystallization was carried out using the sitting drop vapor-diffusion method with an Art Robbins Instruments Phoenix system in the X-ray Crystallography Core Laboratory at UTHSCSA. Crystals were obtained using the following reagents from commercial crystallization screen kits from Qiagen and Molecular Dimensions: sdAb A (concentrated to 12 mg mL^−1^)—25% polyethylene glycol (PEG) 1000, 0.1 M Tris–HCl pH 8.5 at 22°C; sdAb A/NP600 (12 mg mL^−1^)—15% PEG 6000, 5% glycerol at 22°C; sdAb B (11.3 mg mL^−1^) 4.0 M sodium chloride, 0.1 M bicine pH 9 at 22°C; sdAb B/NP600 (12 mg mL^−1^)—20% PEG 4000, 0.16 M ammonium sulfate, 20% glycerol, 0.08 M sodium acetate pH 4.6 at 22°C; sdAb C (12 mg mL^−1^)—30% PEG 550 monomethyl ether/PEG 20000, 0.1 M carboxylic acids mix (sodium formate, ammonium acetate, sodium citrate, sodium/potassium tartrate, sodium oxamate), 0.1 M imidazole/MES pH 6.5 at 4°C; sdAb C/NP632 (12.7 mg mL^−1^)—20% PEG 6000, 0.2 M magnesium chloride, 0.1 M 1,2,3-hexanetriol, 0.1 M sodium acetate pH 5 at 4°C. Crystals were transferred to undersized cryo-loops and manipulated to wick off excess mother liquor prior to flash-cooling in liquid nitrogen. X-ray diffraction data were acquired using a home source Rigaku MicroMax 007HF X-ray Generator equipped with VariMax HR and HF confocal optics and RAXIS–HTC image plate detectors or national synchrotron facilities. Diffraction data were integrated and scaled using XDS (73). The structure of sdAb A was determined by the molecular replacement method implemented in PHASER (74) using a camel single-domain antibody as the search model [Protein Data Bank (PDB) entry 1YC7 (75)]. All other structures were determined using sdAb A as the search model. Coordinates were refined using PHENIX (76), including simulated annealing with torsion angle dynamics, and alternated with manual rebuilding using COOT (77). Non-crystallographic symmetry restraints were used in the refinement of the sdAb B/NP600 and sdAb C/NP632 complexes. Visualizations of structures employed PyMol (78).

## Author Contributions

JG, AT, LS, and AH designed experiments, performed the work, and analyzed the data. PH contributed analytic tools. AH wrote the paper.

## Conflict of Interest Statement

The authors declare that the research was conducted in the absence of any commercial or financial relationships that could be construed as a potential conflict of interest.

## References

[1] Slenczka WG The Marburg virus outbreak of 1967 and subsequent episodes. Curr Top Microbiol Immunol (1999) 235:49–75. 9893378 10.1007/978-3-642-59949-1_4

[2] Towner JS Amman BR Sealy TK Carroll SA Comer JA Kemp A Isolation of genetically diverse Marburg viruses from Egyptian fruit bats. PLoS Pathog (2009) 5:e1000536. 10.1371/journal.ppat.1000536 19649327 PMC2713404

[3] Bausch DG Nichol ST Muyembe-Tamfum JJ Borchert M Rollin PE Sleurs H Marburg hemorrhagic fever associated with multiple genetic lineages of virus. N Engl J Med (2006) 355:909–19. 10.1056/NEJMoa051465 16943403

[4] Towner JS Khristova ML Sealy TK Vincent MJ Erickson BR Bawiec DA Marburgvirus genomics and association with a large hemorrhagic fever outbreak in Angola. J Virol (2006) 80:6497–516. 10.1128/JVI.00069-06 16775337 PMC1488971

[5] Knust B Schafer IJ Wamala J Nyakarahuka L Okot C Shoemaker T Multidistrict outbreak of Marburg virus disease – Uganda, 2012. J Infect Dis (2015) 212(Suppl 2):S119–28. 10.1093/infdis/jiv351 26209681 PMC5649344

[6] Mire CE Geisbert JB Borisevich V Fenton KA Agans KN Flyak AI Therapeutic treatment of Marburg and Ravn virus infection in nonhuman primates with a human monoclonal antibody. Sci Transl Med (2017) 9:eaai8711. 10.1126/scitranslmed.aai8711 28381540 PMC5719873

[7] Grolla A Jones SM Fernando L Strong JE Ströher U Möller P The use of a mobile laboratory unit in support of patient management and epidemiological surveillance during the 2005 Marburg outbreak in Angola. PLoS Negl Trop Dis (2011) 5:e1183. 10.1371/journal.pntd.0001183 21629730 PMC3101190

[8] Bausch DG Schwarz L Outbreak of Ebola virus disease in Guinea: where ecology meets economy. PLoS Negl Trop Dis (2014) 8:e3056 10.1371/journal.pntd.0003056 25079231 PMC4117598

[9] Dietzel E Schudt G Krahling V Matrosovich M Becker S. Functional characterization of adaptive mutations during the West African Ebola virus outbreak. J Virol (2017) 91:e01913–16. 10.1128/JVI.01913-16 27847361 PMC5215343

[10] Kugelman JR Sanchez-Lockhart M Andersen KG Gire S Park DJ Sealfon R Evaluation of the potential impact of Ebola virus genomic drift on the efficacy of sequence-based candidate therapeutics. MBio (2015) 6:e02227–14. 10.1128/mBio.02227-14 25604787 PMC4313914

[11] Gire SK Goba A Andersen KG Sealfon RS Park DJ Kanneh L Genomic surveillance elucidates Ebola virus origin and transmission during the 2014 outbreak. Science (2014) 345:1369–72. 10.1126/science.1259657 25214632 PMC4431643

[12] Sozhamannan S Holland MY Hall AT Negrón DA Ivancich M Koehler JW Evaluation of signature erosion in Ebola virus due to genomic drift and its impact on the performance of diagnostic assays. Viruses (2015) 7:3130–54. 10.3390/v7062763 26090727 PMC4488730

[13] Flyak AI Ilinykh PA Murin CD Garron T Shen X Fusco ML Mechanism of human antibody-mediated neutralization of Marburg virus. Cell (2015) 160:893–903. 10.1016/j.cell.2015.01.031 25723164 PMC4344968

[14] Kajihara M Nakayama E Marzi A Igarashi M Feldmann H Takada A. Novel mutations in Marburg virus glycoprotein associated with viral evasion from antibody mediated immune pressure. J Gen Virol (2013) 94:876–83. 10.1099/vir.0.049114-0 23288419 PMC3709686

[15] Audet J Wong G Wang H Lu G Gao GF Kobinger G Molecular characterization of the monoclonal antibodies composing ZMAb: a protective cocktail against Ebola virus. Sci Rep (2014) 4:6881. 10.1038/srep06881 25375093 PMC5381473

[16] Davidson E Bryan C Fong RH Barnes T Pfaff JM Mabila M Mechanism of binding to Ebola virus glycoprotein by the ZMapp, ZMAb, and MB-003 cocktail antibodies. J Virol (2015) 89:10982–92. 10.1128/JVI.01490-15 26311869 PMC4621129

[17] Kugelman JR Kugelman-Tonos J Ladner JT Pettit J Keeton CM Nagle ER Emergence of Ebola virus escape variants in infected non-human primates treated with the MB-003 antibody cocktail. Cell Rep (2015) 12:2111–20. 10.1016/j.celrep.2015.08.038 26365189

[18] Gong LI Suchard MA Bloom JD. Stability-mediated epistasis constrains the evolution of an influenza protein. Elife (2013) 2:e00631. 10.7554/eLife.00631 23682315 PMC3654441

[19] Lee HK Lee CK Loh TP Chiang D Koay ES Tang JW Missed diagnosis of influenza B virus due to nucleoprotein sequence mutations, Singapore, April 2011. Euro Surveill (2011) 16:19943. 21871229

[20] Goldman ER Anderson GP Liu JL Delehanty JB Sherwood LJ Osborn LE Facile generation of heat-stable antiviral and antitoxin single domain antibodies from a semisynthetic llama library. Anal Chem (2006) 78:8245–55. 10.1021/ac0610053 17165813 PMC2528076

[21] Sherwood LJ Osborn LE Carrion R Jr Patterson JL Hayhurst A. Rapid assembly of sensitive antigen-capture assays for Marburg virus, using *in vitro* selection of llama single-domain antibodies, at biosafety level 4. J Infect Dis (2007) 196(Suppl 2):S213–9. 10.1086/520586 17940952

[22] Bharat TA Riches JD Kolesnikova L Welsch S Krähling V Davey N Cryo-electron tomography of Marburg virus particles and their morphogenesis within infected cells. PLoS Biol (2011) 9:e1001196. 10.1371/journal.pbio.1001196 22110401 PMC3217011

[23] Dolnik O Stevermann L Kolesnikova L Becker S. Marburg virus inclusions: a virus-induced microcompartment and interface to multivesicular bodies and the late endosomal compartment. Eur J Cell Biol (2015) 94:323–31. 10.1016/j.ejcb.2015.05.006 26070789

[24] Mühlberger E Lötfering B Klenk HD Becker S. Three of the four nucleocapsid proteins of Marburg virus, NP, VP35, and L, are sufficient to mediate replication and transcription of Marburg virus-specific monocistronic minigenomes. J Virol (1998) 72:8756–64. 9765419 10.1128/jvi.72.11.8756-8764.1998 PMC110291

[25] Kirchdoerfer RN Moyer CL Abelson DM Saphire EO. The Ebola virus VP30-NP interaction is a regulator of viral RNA synthesis. PLoS Pathog (2016) 12:e1005937. 10.1371/journal.ppat.1005937 27755595 PMC5068707

[26] Sherwood LJ Hayhurst A. Hapten mediated display and pairing of recombinant antibodies accelerates assay assembly for biothreat countermeasures. Sci Rep (2012) 2:807. 10.1038/srep00807 23150778 PMC3495282

[27] Sherwood LJ Hayhurst A. Ebolavirus nucleoprotein C-termini potently attract single domain antibodies enabling monoclonal affinity reagent sandwich assay (MARSA) formulation. PLoS One (2013) 8:e61232. 10.1371/journal.pone.0061232 23577211 PMC3618483

[28] Peng HP Lee KH Jian JW Yang AS. Origins of specificity and affinity in antibody-protein interactions. Proc Natl Acad Sci U S A (2014) 111:E2656–65. 10.1073/pnas.1401131111 24938786 PMC4084487

[29] Hall TA BioEdit: a user-friendly biological sequence alignment editor and analysis program for Windows 95/98/NT. Nucleic Acids Symp Ser (1999) 41:95–8.

[30] Tannous BA Kim DE Fernandez JL Weissleder R Breakefield XO. Codon-optimized Gaussia luciferase cDNA for mammalian gene expression in culture and *in vivo*. Mol Ther (2005) 11:435–43. 10.1016/j.ymthe.2004.10.016 15727940

[31] Venisnik KM Olafsen T Gambhir SS Wu AM. Fusion of Gaussia luciferase to an engineered anti-carcinoembryonic antigen (CEA) antibody for *in vivo* optical imaging. Mol Imaging Biol (2007) 9:267–77. 10.1007/s11307-007-0101-8 17577599

[32] Bharat TA Noda T Riches JD Kraehling V Kolesnikova L Becker S Structural dissection of Ebola virus and its assembly determinants using cryo-electron tomography. Proc Natl Acad Sci U S A (2012) 109:4275–80. 10.1073/pnas.1120453109 22371572 PMC3306676

[33] Hall MP Unch J Binkowski BF Valley MP Butler BL Wood MG Engineered luciferase reporter from a deep sea shrimp utilizing a novel imidazopyrazinone substrate. ACS Chem Biol (2012) 7:1848–57. 10.1021/cb3002478 22894855 PMC3501149

[34] Zhou YH Chen Z Purcell RH Emerson SU. Positive reactions on Western blots do not necessarily indicate the epitopes on antigens are continuous. Immunol Cell Biol (2007) 85:73–8. 10.1038/sj.icb.7100004 17130902

[35] Baker LE Ellena JF Handing KB Derewenda U Utepbergenov D Engel DA Molecular architecture of the nucleoprotein C-terminal domain from the Ebola and Marburg viruses. Acta Crystallogr D Struct Biol (2016) 72:49–58. 10.1107/S2059798315021439 26894534 PMC4905509

[36] Wu M Park YJ Pardon E Turley S Hayhurst A Deng J Structures of a key interaction protein from the *Trypanosoma brucei* editosome in complex with single domain antibodies. J Struct Biol (2011) 174:124–36. 10.1016/j.jsb.2010.10.007 20969962 PMC3037447

[37] Friesen RH Lee PS Stoop EJ Hoffman RM Ekiert DC Bhabha G A common solution to group 2 influenza virus neutralization. Proc Natl Acad Sci U S A (2014) 111:445–50. 10.1073/pnas.1319058110 24335589 PMC3890827

[38] Zhou T Lynch RM Chen L Acharya P Wu X Doria-Rose NA Structural repertoire of HIV-1-neutralizing antibodies targeting the CD4 supersite in 14 donors. Cell (2015) 161:1280–92. 10.1016/j.cell.2015.05.007 26004070 PMC4683157

[39] Kong L Lee JH Doores KJ Murin CD Julien JP McBride R Supersite of immune vulnerability on the glycosylated face of HIV-1 envelope glycoprotein gp120. Nat Struct Mol Biol (2013) 20:796–803. 10.1038/nsmb.2594 23708606 PMC3823233

[40] Sobolev V Eyal E Gerzon S Potapov V Babor M Prilusky J SPACE: a suite of tools for protein structure prediction and analysis based on complementarity and environment. Nucleic Acids Res (2005) 33:W39–43. 10.1093/nar/gki398 15980496 PMC1160159

[41] De Genst E Silence K Decanniere K Conrath K Loris R Kinne J Molecular basis for the preferential cleft recognition by dromedary heavy-chain antibodies. Proc Natl Acad Sci U S A (2006) 103:4586–91. 10.1073/pnas.0505379103 16537393 PMC1450215

[42] Stijlemans B Conrath K Cortez-Retamozo V Van Xong H Wyns L Senter P Efficient targeting of conserved cryptic epitopes of infectious agents by single domain antibodies. African trypanosomes as paradigm. J Biol Chem (2004) 279:1256–61. 10.1074/jbc.M307341200 14527957

[43] Strauss M Schotte L Thys B Filman DJ Hogle JM. Five of five VHHs neutralizing poliovirus bind the receptor-binding site. J Virol (2016) 90:3496–505. 10.1128/JVI.03017-15 26764003 PMC4794687

[44] Krissinel E Henrick K. Inference of macromolecular assemblies from crystalline state. J Mol Biol (2007) 372:774–97. 10.1016/j.jmb.2007.05.022 17681537

[45] Koide A Tereshko V Uysal S Margalef K Kossiakoff AA Koide S. Exploring the capacity of minimalist protein interfaces: interface energetics and affinity maturation to picomolar KD of a single-domain antibody with a flat paratope. J Mol Biol (2007) 373:941–53. 10.1016/j.jmb.2007.08.027 17888451 PMC2148503

[46] Clackson T Wells JA. A hot spot of binding energy in a hormone-receptor interface. Science (1995) 267:383–6. 10.1126/science.7529940 7529940

[47] Bogan AA Thorn KS. Anatomy of hot spots in protein interfaces. J Mol Biol (1998) 280:1–9. 10.1006/jmbi.1998.1843 9653027

[48] Winn MD Ballard CC Cowtan KD Dodson EJ Emsley P Evans PR Overview of the CCP4 suite and current developments. Acta Crystallogr D Biol Crystallogr (2011) 67:235–42. 10.1107/S0907444910045749 21460441 PMC3069738

[49] Epa VC Colman PM Shape and electrostatic complementarity at viral antigen-antibody complexes. Curr Top Microbiol Immunol (2001) 260:45–53. 10.1007/978-3-662-05783-4_3 11443880

[50] Lawrence MC Colman PM. Shape complementarity at protein/protein interfaces. J Mol Biol (1993) 234:946–50. 10.1006/jmbi.1993.1648 8263940

[51] Rini JM Schulze-Gahmen U Wilson IA. Structural evidence for induced fit as a mechanism for antibody-antigen recognition. Science (1992) 255:959–65. 10.1126/science.1546293 1546293

[52] Kallewaard NL Corti D Collins PJ Neu U McAuliffe JM Benjamin E Structure and function analysis of an antibody recognizing all influenza A subtypes. Cell (2016) 166:596–608. 10.1016/j.cell.2016.05.073 27453466 PMC4967455

[53] Hinz A Lutje Hulsik D Forsman A Koh WW Belrhali H Gorlani A Crystal structure of the neutralizing llama V(HH) D7 and its mode of HIV-1 gp120 interaction. PLoS One (2010) 5:e10482. 10.1371/journal.pone.0010482 20463957 PMC2864739

[54] Yusim K Yoon H Foley B Feng S Macke J Dimitrijevic M Integrated sequence and immunology filovirus database at Los Alamos. Database (Oxford) (2016) 2016. 10.1093/database/baw047 27103629 PMC4839628

[55] Saijo M Niikura M Maeda A Sata T Kurata T Kurane I Characterization of monoclonal antibodies to Marburg virus nucleoprotein (NP) that can be used for NP-capture enzyme-linked immunosorbent assay. J Med Virol (2005) 76:111–8. 10.1002/jmv.20332 15778962

[56] Ma B Elkayam T Wolfson H Nussinov R. Protein-protein interactions: structurally conserved residues distinguish between binding sites and exposed protein surfaces. Proc Natl Acad Sci U S A (2003) 100:5772–7. 10.1073/pnas.1030237100 12730379 PMC156276

[57] Van Regenmortel MH. The antigenicity of tobacco mosaic virus. Philos Trans R Soc Lond B Biol Sci (1999) 354:559–68. 10.1098/rstb.1999.0407 10212935 PMC1692532

[58] Holm L Laakso LM. Dali server update. Nucleic Acids Res (2016) 44:W351–5. 10.1093/nar/gkw357 27131377 PMC4987910

[59] Dziubanska PJ Derewenda U Ellena JF Engel DA Derewenda ZS. The structure of the C-terminal domain of the *Zaire ebolavirus* nucleoprotein. Acta Crystallogr D Biol Crystallogr (2014) 70:2420–9. 10.1107/S1399004714014710 25195755 PMC4157450

[60] Drozdetskiy A Cole C Procter J Barton GJ. JPred4: a protein secondary structure prediction server. Nucleic Acids Res (2015) 43:W389–94. 10.1093/nar/gkv332 25883141 PMC4489285

[61] Liu B Dong S Li G Wang W Liu X Wang Y Structural insight into nucleoprotein conformation change chaperoned by VP35 peptide in Marburg virus. J Virol (2017) 91:e00825–17. 10.1128/JVI.00825-17 28566377 PMC5533893

[62] Koide S. Engineering of recombinant crystallization chaperones. Curr Opin Struct Biol (2009) 19:449–57. 10.1016/j.sbi.2009.04.008 19477632 PMC2736338

[63] Noda T Watanabe S Sagara H Kawaoka Y. Mapping of the VP40-binding regions of the nucleoprotein of Ebola virus. J Virol (2007) 81:3554–62. 10.1128/JVI.02183-06 17229682 PMC1866061

[64] Lee PS Wilson IA. Structural characterization of viral epitopes recognized by broadly cross-reactive antibodies. Curr Top Microbiol Immunol (2015) 386:323–41. 10.1007/82_2014_413 25037260 PMC4358778

[65] Xu R Krause JC McBride R Paulson JC Crowe JE Jr Wilson IA. A recurring motif for antibody recognition of the receptor-binding site of influenza hemagglutinin. Nat Struct Mol Biol (2013) 20:363–70. 10.1038/nsmb.2500 23396351 PMC3594569

[66] Oda S Noda T Wijesinghe KJ Halfmann P Bornholdt ZA Abelson DM Crystal structure of Marburg virus VP40 reveals a broad, basic patch for matrix assembly and a requirement of the N-terminal domain for immunosuppression. J Virol (2015) 90:1839–48. 10.1128/JVI.01597-15 26656687 PMC4733994

[67] Welsch S Kolesnikova L Krähling V Riches JD Becker S Briggs JA. Electron tomography reveals the steps in filovirus budding. PLoS Pathog (2010) 6:e1000875. 10.1371/journal.ppat.1000875 20442788 PMC2861712

[68] Cleveland SB Davies J McClure MA. A bioinformatics approach to the structure, function, and evolution of the nucleoprotein of the order mononegavirales. PLoS One (2011) 6:e19275. 10.1371/annotation/6e05f8a1-c49a-4102-a8c9-188e8dc6290e 21559282 PMC3086907

[69] Neu HC Heppel LA The release of enzymes from *Escherichia coli* by osmotic shock and during the formation of spheroplasts. J Biol Chem (1965) 240:3685–92. 4284300

[70] Austin BP Nallamsetty S Waugh DS. Hexahistidine-tagged maltose-binding protein as a fusion partner for the production of soluble recombinant proteins in *Escherichia coli*. Methods Mol Biol (2009) 498:157–72. 10.1007/978-1-59745-196-3_11 18988025

[71] Center RJ Kobe B Wilson KA Teh T Howlett GJ Kemp BE Crystallization of a trimeric human T cell leukemia virus type 1 gp21 ectodomain fragment as a chimera with maltose-binding protein. Protein Sci (1998) 7:1612–9. 10.1002/pro.5560070715 9684894 PMC2144054

[72] Sadler JR Sasmor H Betz JL. A perfectly symmetric lac operator binds the lac repressor very tightly. Proc Natl Acad Sci U S A (1983) 80:6785–9. 10.1073/pnas.80.22.6785 6316325 PMC390070

[73] Kabsch W. XDS. Acta Crystallogr D Biol Crystallogr (2010) 66:125–32. 10.1107/S0907444909047337 20124692 PMC2815665

[74] McCoy AJ Grosse-Kunstleve RW Adams PD Winn MD Storoni LC Read RJ. Phaser crystallographic software. J Appl Crystallogr (2007) 40:658–74. 10.1107/S0021889807021206 19461840 PMC2483472

[75] Conrath K Vincke C Stijlemans B Schymkowitz J Decanniere K Wyns L Antigen binding and solubility effects upon the veneering of a camel VHH in framework-2 to mimic a VH. J Mol Biol (2005) 350:112–25. 10.1016/j.jmb.2005.04.050 15913651

[76] Adams PD Afonine PV Bunkóczi G Chen VB Davis IW Echols N PHENIX: a comprehensive python-based system for macromolecular structure solution. Acta Crystallogr D Biol Crystallogr (2010) 66:213–21. 10.1107/S0907444909052925 20124702 PMC2815670

[77] Emsley P Lohkamp B Scott WG Cowtan K. Features and development of coot. Acta Crystallogr D Biol Crystallogr (2010) 66:486–501. 10.1107/S0907444910007493 20383002 PMC2852313

[78] DeLano WL The PyMol Molecular Graphics System. San Carlos, CA: deLano Scientific (2002). Available from: http://www.pymol.org

