# Supplementary material for: Unveiling a Drift Resistant Cryptotope within Marburgvirus Nucleoprotein Recognized by Llama Single-Domain Antibodies
Source: Front Immunol. 2017 Oct 2;8:1234. doi: 10.3389/fimmu.2017.01234 (PMC5630700; doi:10.3389/fimmu.2017.01234)
Supplement: Supplementary file 1 [file Presentation_1.PDF]

Supplementary Material

Unveiling a drift resistant cryptotope within *Marburgvirus* nucleoprotein recognized by llama single domain antibodies

John Anthony Garza, Alexander Bryan Taylor, Laura Jo Sherwood, Peter John Hart, Andrew Hayhurst*

*** Correspondence:** Corresponding Author: ahayhurst@TxBiomed.org

Supplementary Figure 1.

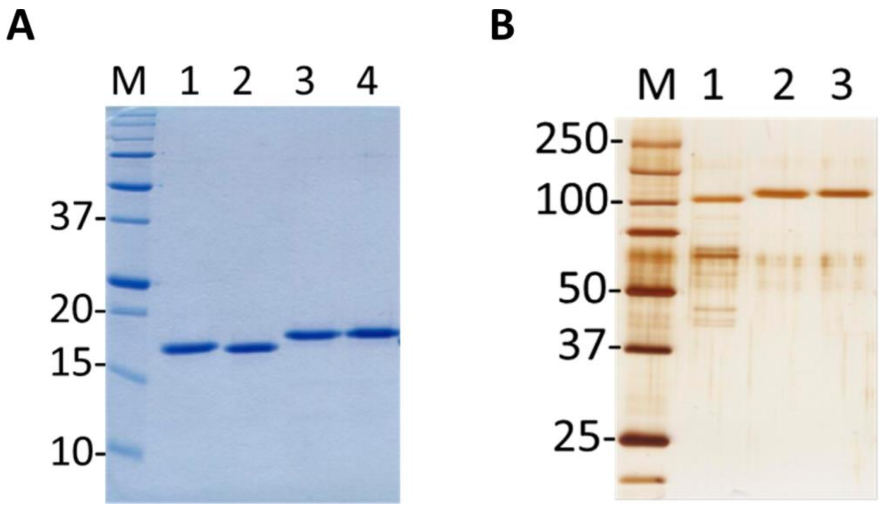

sdAb and NP components employed in the NP sandwich assay. A. Coomassie stained SDS-PAGE analysis of sdAb derived from pecan22 periplasmic expression vector following IMAC and SEC with 2 µg of protein loaded per lane; M, molecular weight markers with sizes in kDa; 1, sdAb A; 2, sdAb B; 3, sdAb C; 4, sdAb D. **B.** Silver stained SDS-PAGE analysis of CsCl gradient purified recombinant NP proteins with 250 ng loaded; 1, MARV Musoke; 2, EBOV Zaire; 3, Ebola Bundibugyo; M, molecular weight markers with sizes in kDa.

Supplementary Figure 2.

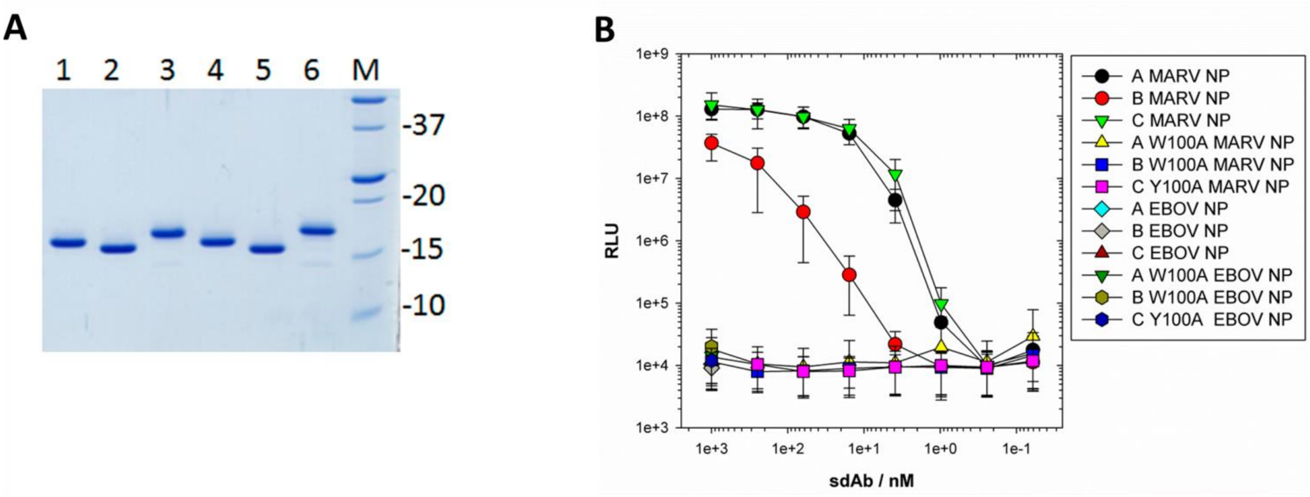

Pivotal nature of central CDR3 aromatic residue. A. Coomassie stained SDS-PAGE of 2 µg of wild type pecan126 derived sdAb and Ala100 mutants; 1, sdAb A; 2, sdAb B; 3, sdAb C; 4, sdAb A Trp100Ala; 5, sdAb B Trp100Ala; sdAb C, Tyr100Ala; M, molecular weight markers with sizes in kDa. **B.** Purified wild-type sdAb or alanine substituted sdAb were titrated in duplicate dilutions over constant amounts of recombinant MARV or EBOV NP and captured sdAb revealed with anti-His HRP conjugate. The experiment was repeated once and the plot is the mean of the two experiments with error bars representing +/- standard deviation.

Supplementary Figure 3.

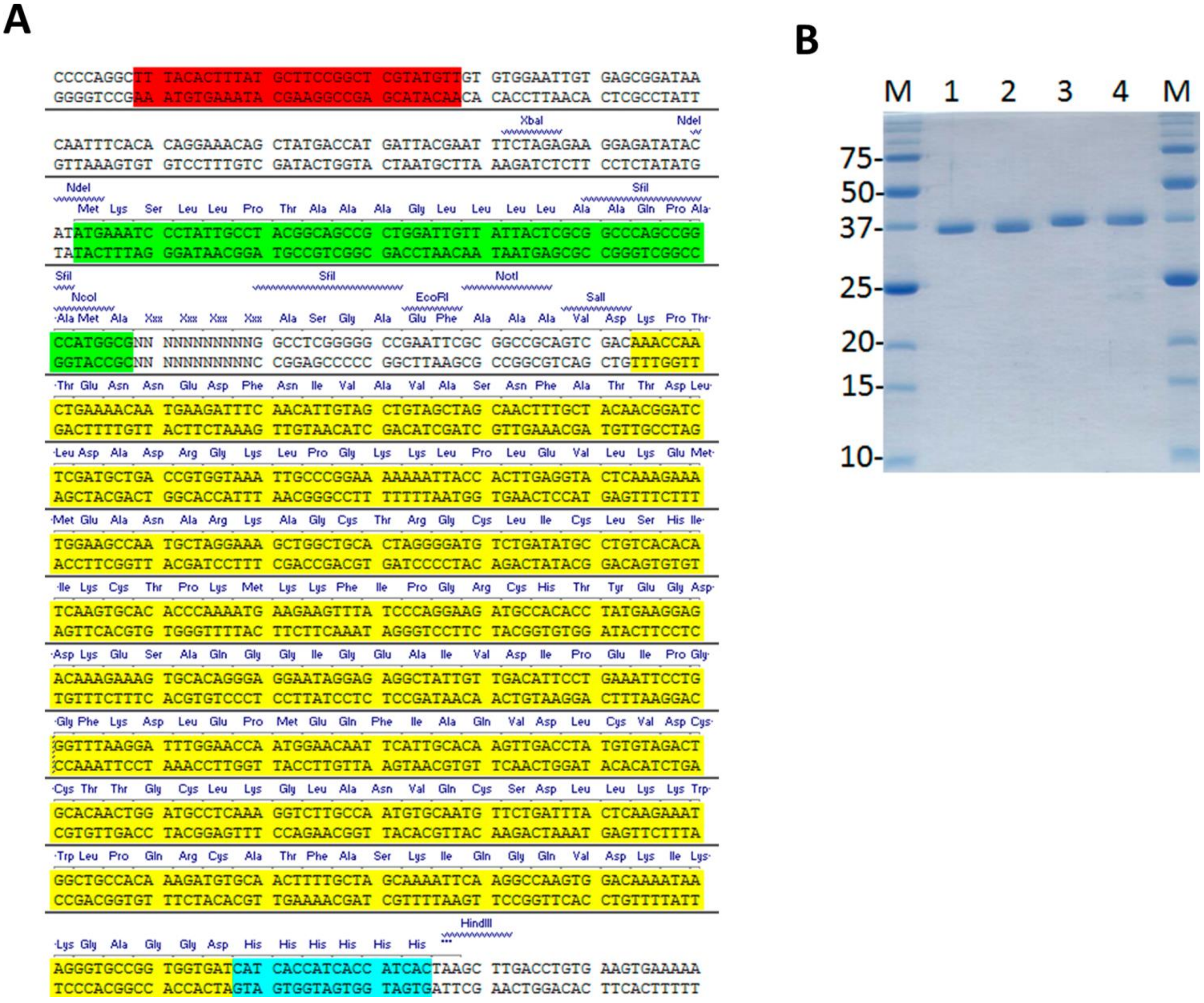

sdAb-gluc fusion protein (glucibody) production. A. Excerpt of the parental pecan35 sequence where a *lac* promoter (red) drives *pel*B leader (green) based secretion of the synthetic mature gluc gene (yellow) that has a His_6 tag at the C-terminus (light blue). The polylinker is occupied with various stuffers indicated as XXX to allow most recombinant antibody fragment designs to be easily inserted *via Nco*I/*Not*I, *Sfi*I/*Not*I or *Sfi*I/*Sfi*I. **B.** Coomassie stained SDS-PAGE of pecan35 derived glucibodies purified from the periplasm using IMAC and SEC with 1 µg of each fusion protein loaded; M, molecular weight markers with sizes indicated in kDa; 1, sdAb A glucibody; 2, sdAb B glucibody; 3, sdAb C glucibody; 4, sdAb D glucibody.

Supplementary Figure 4.

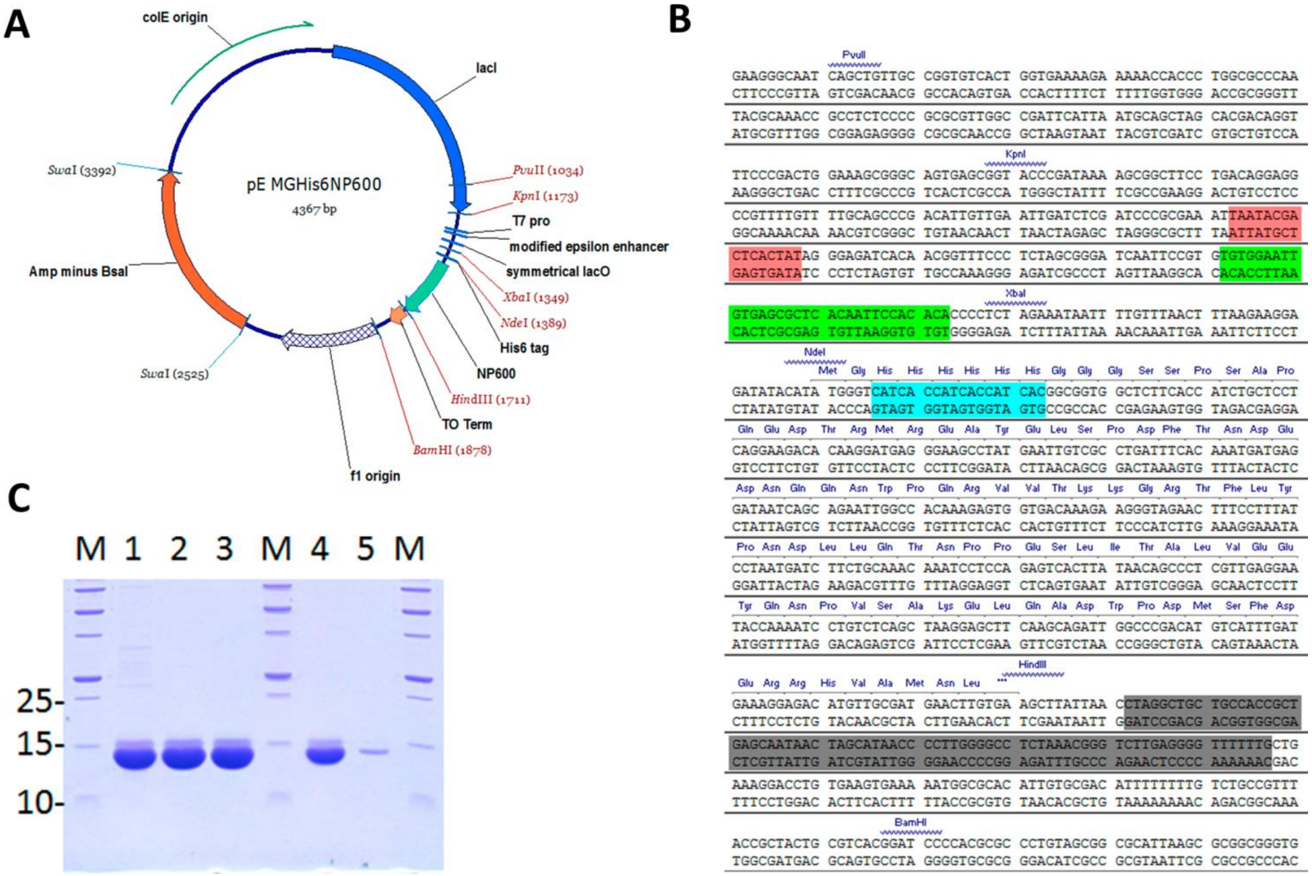

Production of NP600. A. Map of cytosolic expression vector pE-MGHis6NP600 for expressing the MARV C-terminal domain NP600 used for Western blotting of sdAb A, B and C and for crystal complex formation with sdAb A and B. **B.** The sequence of the T7 expression cassette is shown with highlights as follows; T7 promoter (T7 pro), red; symmetrical lac operator (lacO), green; His6 tag, light blue; major T7 terminator (TØ), grey. **C.** Coomassie stained SDS-PAGE gel of various stages in the purification of NP600; 1, IMAC elution, 5 µL; 2, Q-sepharose elution, 5 µL; S75 elution, 5 µL; M, markers; 4, post quantification, 10 µg; 5, post quantification, 1µg.

Supplementary Figure 5.

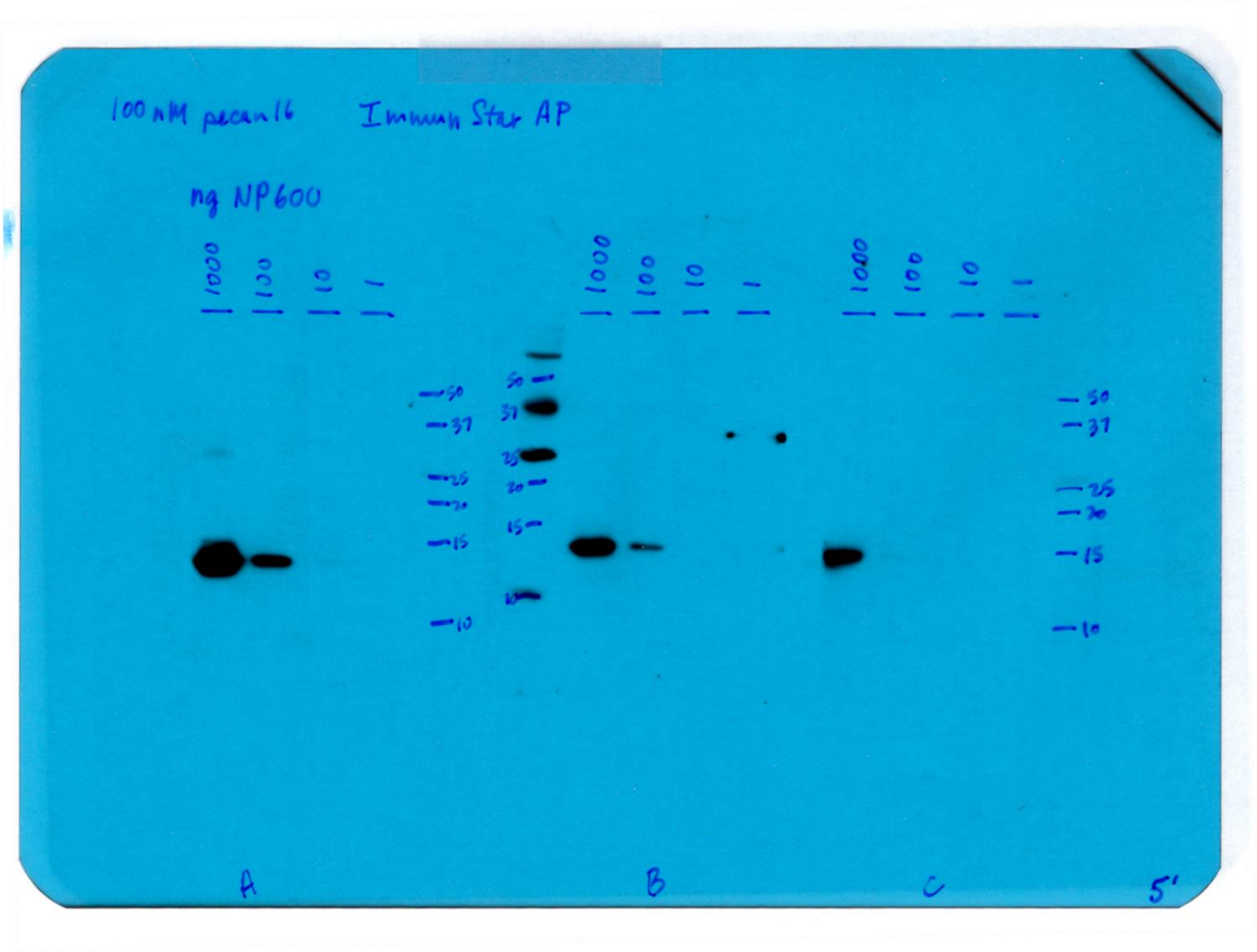

Original Western blot exposure used to generate the collage in figure 1E. Tenfold serial dilutions of purified NP600 were separately probed with 100 nM sdAb-AP fusion proteins A, B and C and the blots subsequently exposed to a single film for 5 min.

Supplementary Figure 6.

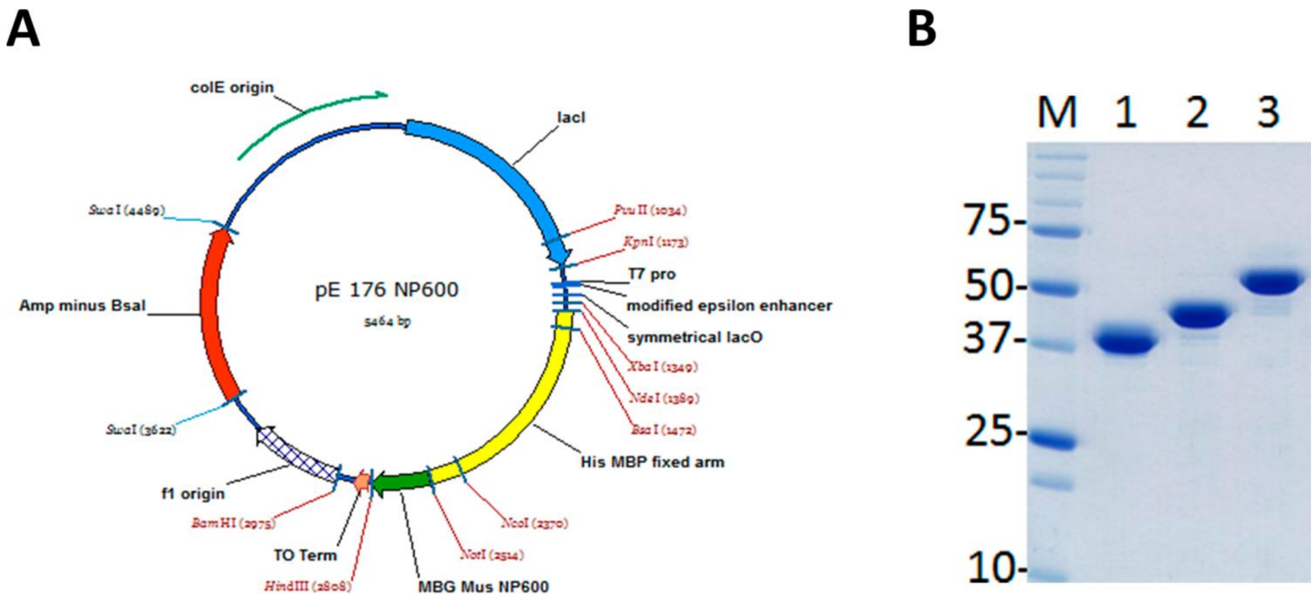

Maltose binding protein fusions. A. Expression vector pE generating N-terminally His_6 tagged MBP fusion proteins which is NP600 in this example. **B.** Coomassie stained SDS-PAGE analysis of purified mbp proteins with 5 µg loaded per lane; M, molecular weight markers with sizes indicated in kDa; 1, mbp control; 2, mbp-NP632; 3, mbp-NP600.

Supplementary Figure 7.

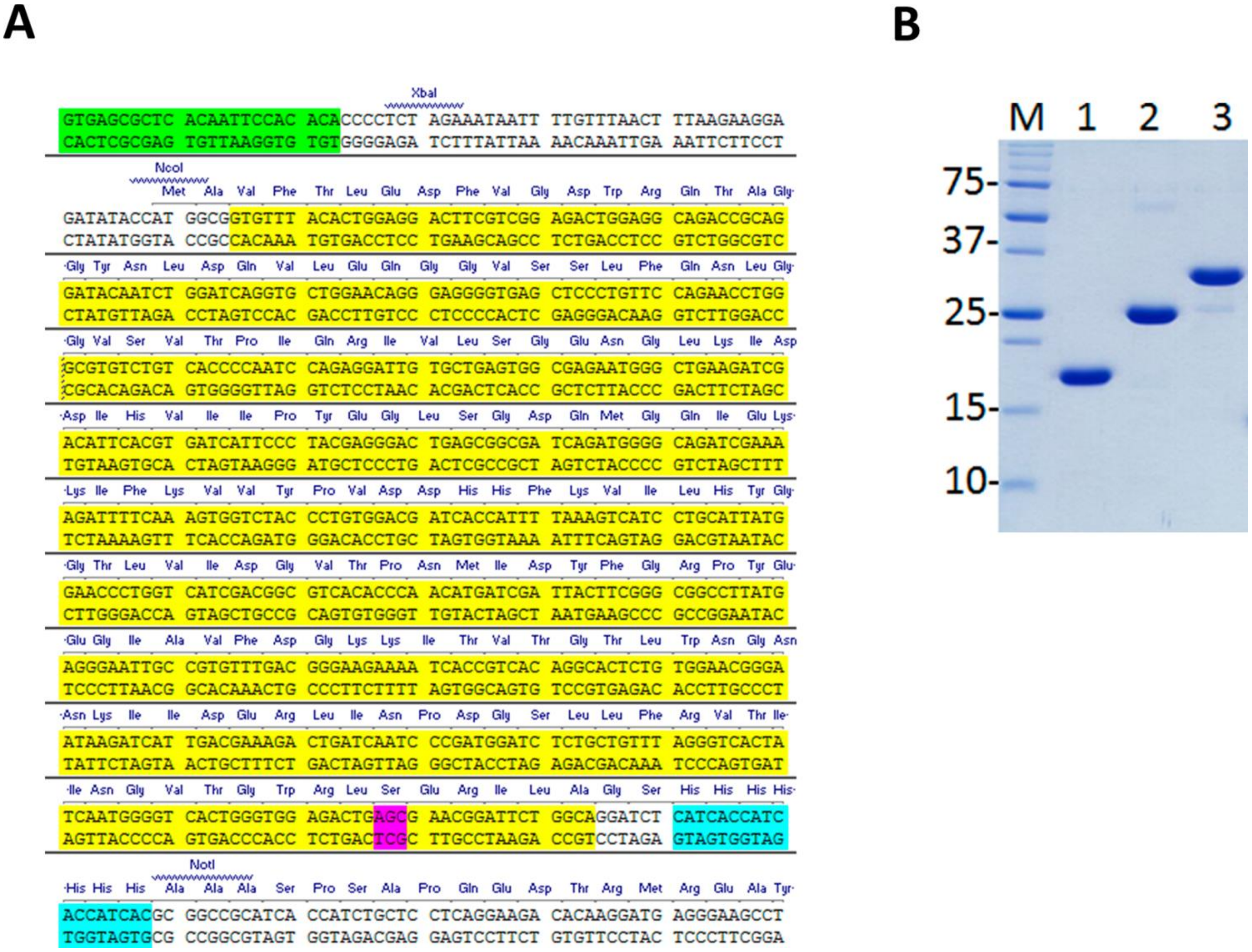

Nluc-NP600 and NP632 fusion protein production. A. Sequence of synthetic nanoluciferase gene within pENCO9 in the same T7 promoter/terminator configuration as pE in supplementary figure 4A except the initiation codon is within a rebuilt translation initiation region as part of an *Nco*I restriction site. The Ser substitution of the Cys within nluc is highlighted in pink, the His_6 tag in turquoise and part of the symmetrical lac operator is shown in green. The His_6 stretch lies between the antigen of interest, here a portion of NP600. **B.** Coomassie stained SDS-PAGE of 2 µg of pure protein; 1, nluc; 2, nluc-NP632; 3, nluc-NP600. M are molecular weight markers with sizes indicated in kDa.

Supplementary Figure 8.

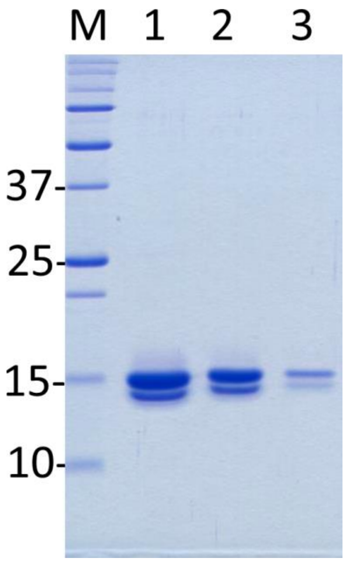

Prey-bait strategy yielding sdAb C/NP632 complex. Coomassie stained gel of the final purified complex used for crystallization; 1, 10 µg; 2, 5 µg; 3, 2.5 µg; M, molecular weight markers with sizes indicated in kDa

Supplementary Figure 9.

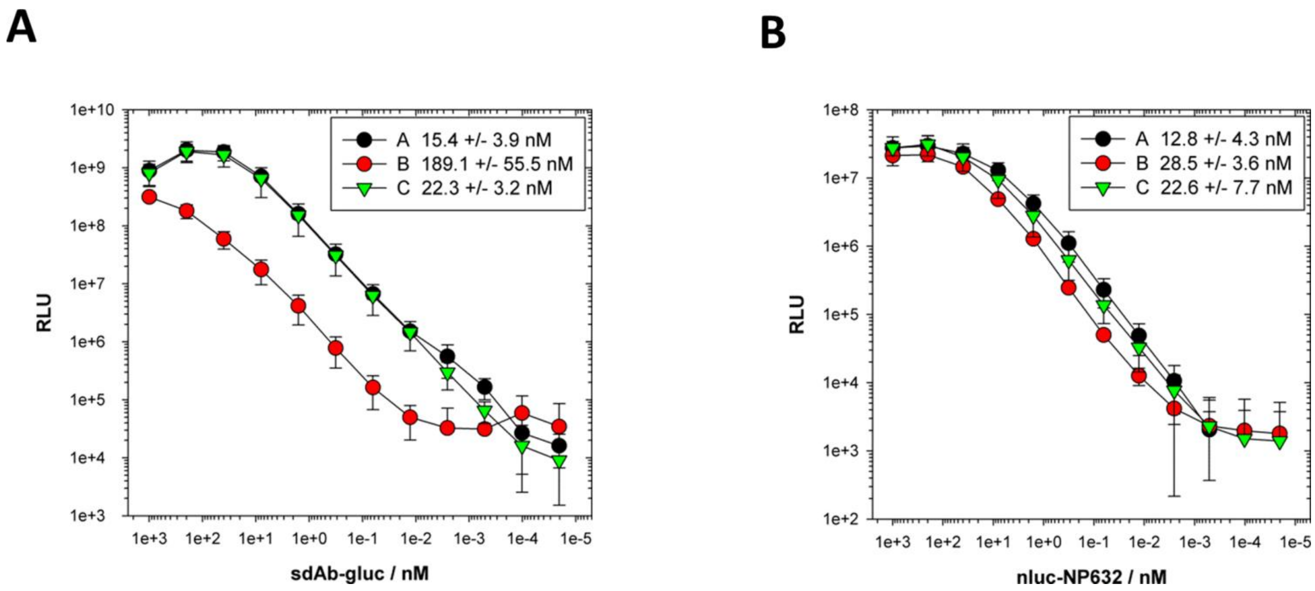

EC_{50} determination for sdAb interacting with NP632 rather than NP600. A. Glucibodies were titrated over passively immobilized mbp-NP632 fusion protein - each titration was performed in duplicate wells with a negative control mbp only binding curve subtracted from each mbp-NP632 curve. The experiment was repeated three times and the plots represent the mean values with error bars +/- standard deviation. The EC_{50} values were determined for each curve and are shown in the legend for each sdAb-gluc fusion protein +/- standard deviation. **B.** Fusions of nluc-NP632 were titrated over oriented immobilized monovalent sdAb - each titration was performed in duplicate wells with a negative control nluc only binding curve subtracted from each nluc-NP600 curve. The experiment was repeated three times and the plots represent the mean values with error bars +/- standard deviation. The EC_{50} values were determined for each curve and are shown in the legend for each sdAb +/- standard deviation.

Supplementary Figure 10.

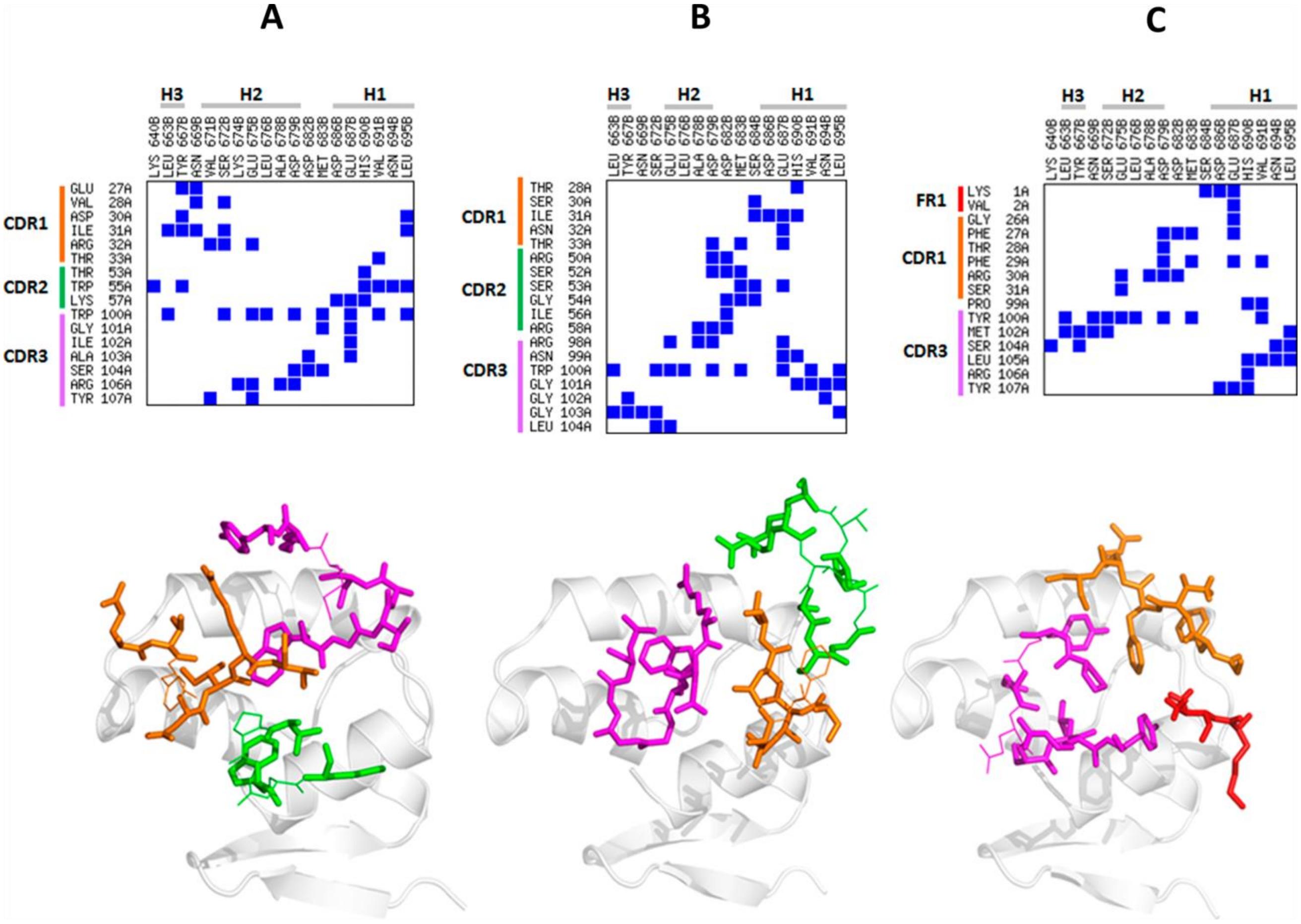

Contact mapping analysis reveals strikingly different CDR usages to engage NP. Top. **C**oordinate PDB files were submitted to the Weizmann server http://ligin.weizmann.ac.il/cma/ with zero threshold for surface area to highlight all potential contacts for sdAb A (**A)** sdAb B (**B)** and sdAb C (**C**) complexes. The alpha helices comprising the epitope are labeled (H) along the top of each contact map with sdAb residues group down the left hand side. The apical CDR3 aromatic residue at position100 is noteworthy with 9 potential contacts shared between sdAb A and B, 7 of which are also shared by sdAb C. **Bottom.** Each sdAb is visualized with CDR loops only (1, orange; 2, green; 3, purple) and the start of FR1 (red) for sdAb C looking down on the epitope (white cartoon) with sdAb contacts shown as sticks and non-contacts shown as lines.

Supplementary Figure 11.

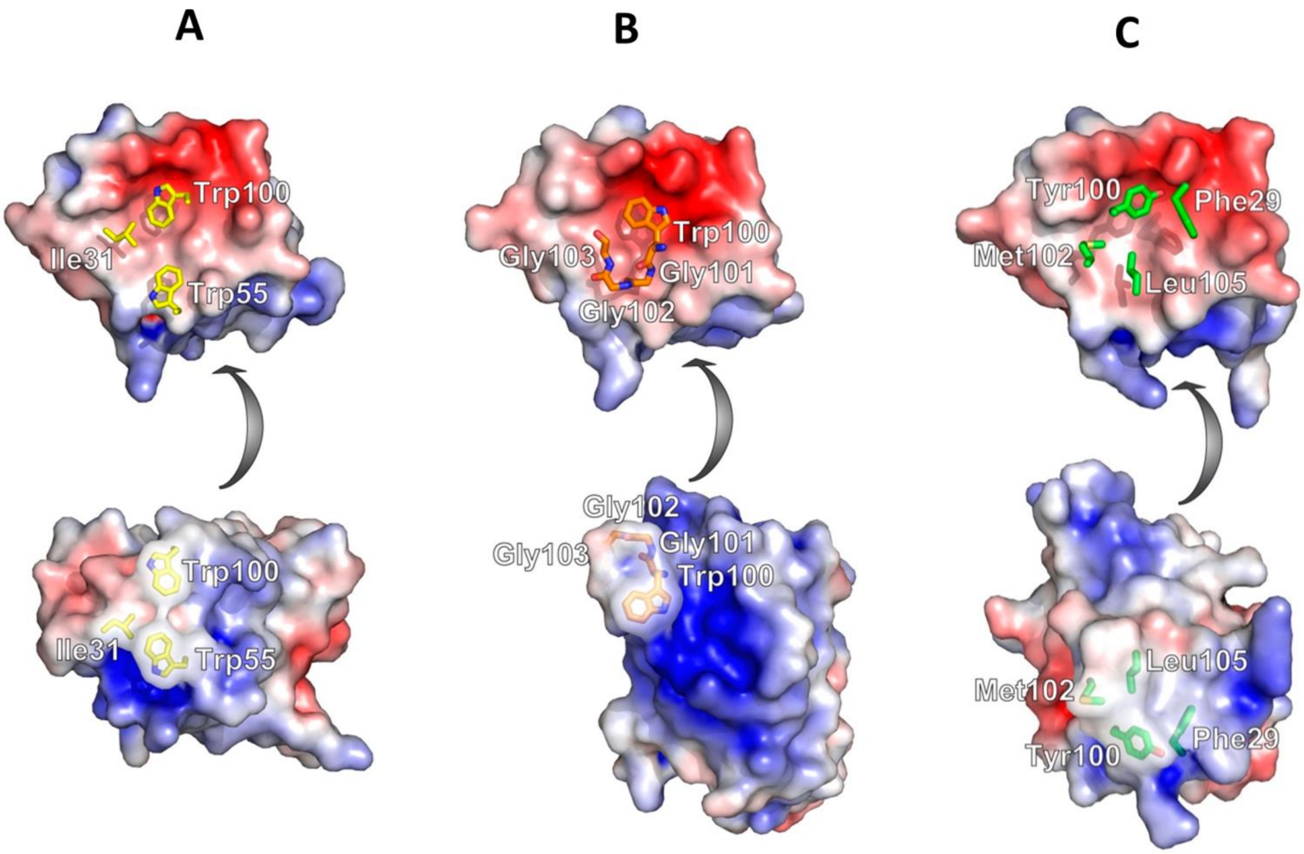

Alternative view of sdAb docking to antigen. Stick form representations of only the basin occupying sdAb side-chains are shown in the basin of the antigen (upper) with the sdAb (lower) pulled out and rotated 180° towards the reader. **A**, sdAb A; **B**, sdAb B; **C**, sdAb C.
